# Defeating depolarizing fields with artificial flux closure in ultrathin ferroelectrics

**DOI:** 10.1038/s41563-023-01674-2

**Published:** 2023-10-02

**Authors:** Elzbieta Gradauskaite, Quintin N. Meier, Natascha Gray, Martin F. Sarott, Tizian Scharsach, Marco Campanini, Thomas Moran, Alexander Vogel, Karla Del Cid-Ledezma, Bryan D. Huey, Marta D. Rossell, Manfred Fiebig, Morgan Trassin

**Affiliations:** 1 Department of Materials, ETH Zurich, Zurich, Switzerland; 2 Univ. Grenoble Alpes, CEA, LITEN, DEHT, Grenoble, France; 3 Electron Microscopy Center, Empa, Dübendorf, Switzerland; 4 Department of Materials Science and Engineering, University of Connecticut, Storrs, CT USA

**Keywords:** Ferroelectrics and multiferroics, Surfaces, interfaces and thin films, Topological defects

## Abstract

Material surfaces encompass structural and chemical discontinuities that often lead to the loss of the property of interest in so-called dead layers. It is particularly problematic in nanoscale oxide electronics, where the integration of strongly correlated materials into devices is obstructed by the thickness threshold required for the emergence of their functionality. Here we report the stabilization of ultrathin out-of-plane ferroelectricity in oxide heterostructures through the design of an artificial flux-closure architecture. Inserting an in-plane-polarized ferroelectric epitaxial buffer provides the continuity of polarization at the interface; despite its insulating nature, we observe the emergence of polarization in our out-of-plane-polarized model of ferroelectric BaTiO_3_ from the very first unit cell. In BiFeO_3_, the flux-closure approach stabilizes a 251° domain wall. Its unusual chirality is probably associated with the ferroelectric analogue to the Dzyaloshinskii–Moriya interaction. We, thus, see that in an adaptively engineered geometry, the depolarizing-field-screening properties of an insulator can even surpass those of a metal and be a source of functionality. This could be a useful insight on the road towards the next generation of oxide electronics.

## Abstract (web summary)

Ferroelectric dead layers can form at perovskite interfaces—a major challenge in integrating oxide thin films into devices. Here, by depositing an in-plane-polarized epitaxial buffer layer of Bi_5_FeTi_3_O_15_, out-of-plane polarization is demonstrated in ultrathin films down to the single-unit-cell level.

## Main

Uncompensated bound charges at the surfaces of ferroelectric films trigger a depolarizing field^1^. It is oriented opposite to the spontaneous polarization and highest when this polarization is normal to the surface—the preferred orientation for ferroelectric devices. Thus, the ferroelectric properties are attenuated with decreasing thickness and completely disappear below about 5 unit cells (u.c.) in most perovskites^2,3^. This holds true even when ferroelectrics are deposited on metallic electrodes, all of which have a finite screening length and normally cannot fully compensate for the bound charges^4^. To maintain polarization in the ultrathin regime, attempts have been made to minimize the surface-charge accumulation or to enforce polar displacements originating from the ferroelectric–electrode interface via interface engineering. For instance, interface chemistry can be tailored to create a favourable charge-screening environment and hence net polarization in the ultrathin regime^5^. Polar metals were also suggested to eliminate the critical thickness in ferroelectrics by inducing inversion symmetry breaking from the bottom interface^6^, but their implementation remains elusive due to the scarcity of lattice-matching systems.

The interfacial polarization discontinuity can be avoided with the use of in-plane-polarized ferroelectrics, which are less susceptible to the depolarizing field even at low thickness^7,8^. In-plane polarization is, however, incompatible with the coveted state-of-the-art, energy-efficient out-of-plane capacitor device geometry. Thus, the mutually exclusive benefits of out-of-plane and in-plane-polarized ferroelectrics pose a serious obstacle to the ongoing quest for the next generation of oxide electronics^9,10^.

Here we combine the ‘best of both worlds’ by eliminating the interfacial polar discontinuity in an out-of-plane-polarized ferroelectric heterostructure by creating a flux-closure-like^11,12^ domain architecture. We accomplish this by interfacing two of the most debated out-of-plane-polarized systems, namely, BaTiO_3_ (BTO) and BiFeO_3_ (BFO), with an in-plane-polarized layered ferroelectric of the Aurivillius phase^13^, that is, Bi_5_FeTi_3_O_15_ (BFTO). In typical perovskite heterostructures, the substrate lattice sets the polarization anisotropy of the epitaxial heterostructure. In our case, the integration of in-plane-polarized BFTO with out-of-plane-polarized systems provides polarization continuity at the interface and enables the onset of an out-of-plane ferroelectric polarization from the very first unit cell. The functional impact of the flux-closure-like ferroelectric interface is highlighted in two ways. First, we demonstrate local switching of the BTO polarization, despite the absence of a conducting bottom electrode. Second, we stabilize homochiral polar Néel domain walls in multiferroic BFO thin films. We, thus, introduce ferroelectric heterostructures with perpendicular in-plane and out-of-plane polar anisotropies for the design of electric polarization at the nanoscale, offering a superior alternative to metal-based device paradigms for ferroelectrics.

### Enforcing out-of-plane polarization from the first unit cell

**Fig. 1:**
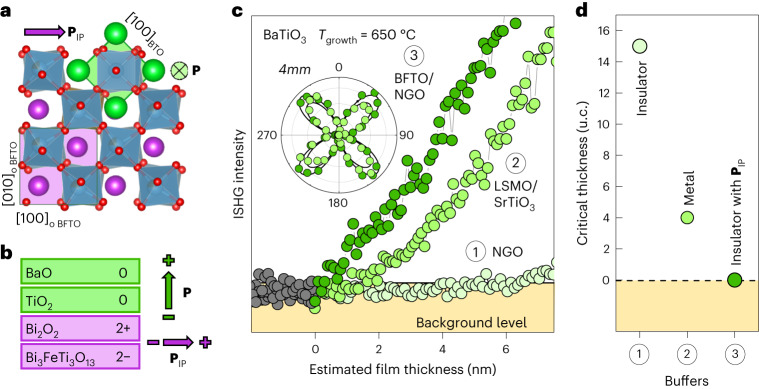
Absence of critical thickness for ferroelectricity in BTO grown on 0.5 u.c. BFTO. **a**, Visualization of the epitaxial relationship between BFTO and BTO unit cells in the plane of the films. **b**, Atomic-plane stacking at the BTO–BFTO interface, showing the upward BTO polarization direction set by the net-positive layer charge at the interface. **c**, ISHG signal tracking of the BTO thin-film polarization during identical deposition on (1) insulating NGO (001)_o_, (2) metallic LSMO on SrTiO_3_ (001) and (3) 0.5 u.c. BFTO on NGO (001)_o_. The inset shows SHG polarimetry scans of the ISHG signal after BTO growth completion on (2) and (3). The polarization of the incoming light is varied from 0° to 360° and the outgoing light is detected at 0°. The black line shows the fit to the SHG signal permitted for the 4*mm* point-group symmetry of BTO. The grey circles correspond to the background ISHG signal prior to the deposition of the BTO layers. **d**, Dependence of critical thickness for ferroelectricity in BTO thin films on the three sample configurations in **c**.

We begin our investigation by monitoring the emergence of polarization in a BTO film epitaxially grown on an in-plane-polarized BFTO buffer layer, using optical second-harmonic generation (SHG). This process is sensitive to inversion symmetry breaking and therefore an ideal probe for ferroelectricity. When measured during the pulsed-laser-deposition process, with in situ second-harmonic generation (ISHG)^3^ (Methods) one directly accesses the emergence of polarization in ultrathin films with unit-cell accuracy owing to simultaneous monitoring by reflection high-energy electron diffraction. We select the Aurivillius compound BFTO as our model in-plane-polarized ferroelectric buffer because it can be grown with single-crystal quality on NdGaO_3_ (NGO) (001)_o_ (refs. ^8,14^) (‘o’ denotes an orthorhombic lattice) and exhibits a robust in-plane polarization from the first half of the unit cell^8^. Furthermore, BFTO is structurally compatible with functional perovskite oxides (Fig. 1a), and its charged layered architecture (layer polarization^15^) can be used to stabilize and orient the out-of-plane polarization^3^ in ferroelectrics grown on top of it (Supplementary Note 1). For instance, BFTO favours an upward-pointing polarization in a BTO layer grown on top (Fig. 1b).

The absence of an ISHG signal and hence spontaneous polarization during the early stage of the direct growth of (001)-oriented BTO on the insulating NGO substrate (Fig. 1c) confirms the dominant role of the depolarizing field in a non-charge-screening environment. Only after passing the thickness of 15 u.c., we detect a signal indicating an out-of-plane-oriented spontaneous polarization. The conventional approach to combat the depolarizing field and reduce the critical thickness involves the use of a lattice-matching conducting buffer such as La_0.7_Sr_0.3_MnO_3_ (LSMO). Indeed, the associated conduction leads to a substantial improvement in charge screening. ISHG measurements (Fig. 1c) reveal a reduction in the thickness threshold to 4 u.c. for the onset of ferroelectricity; however, it does not completely eradicate it. We, therefore, replace the metal with 0.5 u.c. of ferroelectric in-plane-polarized BFTO. Note that the first half of the BFTO unit cell (~2 nm in height) already contains four perovskite layers, which give rise to the net in-plane polarization and exhibit the full functionality of the Aurivillius compound^8,16^. At first glance, this substitution seems counterproductive because of the insulating nature of this polar buffer. However, Fig. 1c shows that we obtain an ISHG signal with the very first BTO unit cell deposited on top of the BFTO; the film grows right in the ferroelectric phase, without any critical thickness. The subsequent continuous ISHG increase with the ongoing BTO deposition is consistent with an out-of-plane-polarized ferroelectric single-domain configuration^3^, characteristic of BTO. SHG polarimetry (Fig. 1c, inset) can be attributed to the tetragonal 4*mm* point-group symmetry of BTO and hence confirms the ferroelectric origin of the ISHG signal. Ex situ X-ray diffraction studies confirm the presence of (001)-oriented BTO with the expected epitaxial relationship, involving a 45° in-plane rotation of the BTO unit cell with respect to the orthorhombic unit cell of the BFTO buffer (Supplementary Note 2). We, thus, see that an in-plane-polarized insulating buffer layer can surpass metallic electrodes in stabilizing an out-of-plane-polarized ferroelectric state in the ultrathin regime (Fig. 1d).

### Metal-free poling in flux-closing heterostructures

**Fig. 2:**
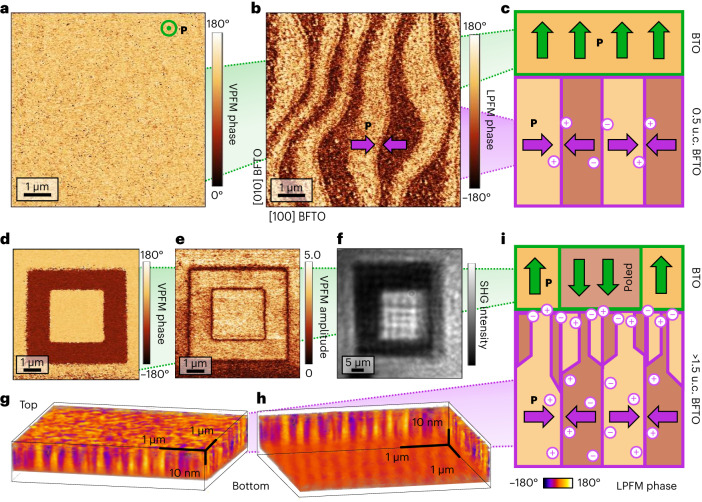
Ferroelectric domain configuration in BTO–BFTO heterostructures. **a**,**b**, VPFM (**a**) and LPFM (**b**) phase scans of the BTO–BFTO bilayer reveal a uniform out-of-plane polarization in BTO and a stripe pattern of in-plane-polarized domains in BFTO. **c**, Sketch of the domain configuration formed by the two layers resulting from **a** and **b**, creating a partial flux-closure architecture. **d**,**e**, VPFM phase (**d**) and amplitude (**e**) scans confirming local polarization reversal in BTO (10 nm) grown on 3.5 u.c. BFTO without a metal electrode. **f**, SHG imaging of a 30 × 30 μm^2^-poled area confirms the ferroelectric nature of the switch. **g**,**h**, Three-dimensional PFM tomographic reconstructions of in-plane-polarized domains in a 5-u.c.-thick BFTO film, viewed obliquely from above (**g**) and below (**h**), directly revealing a distinct stripe and smaller, columnar domain regimes. **i**, Representation of the thickness-dependent domain architecture in BFTO. The higher thickness results in an increased density of mobile charges at the interface, which emulates an electrode and thus enables polarization switching.

To shed light on this seemingly counterintuitive observation, we investigate the ferroelectric domain configuration in our BTO–BFTO bilayer by piezoresponse force microscopy (PFM). The uniform vertical PFM (VPFM) contrast (Fig. 2a) is consistent with an out-of-plane-polarized single-domain configuration in the top BTO layer of 10 nm thickness as inferred from both ISHG and X-ray diffraction. The lateral PFM (LPFM) phase scan (Fig. 2b; Supplementary Fig. 3 shows the LPFM amplitude), however, reveals a pattern of 200-nm-wide stripe domains with alternating tail-to-tail (TT) and head-to-head (HH) charged domain walls (CDWs) characteristic of the buried, fully in-plane-polarized BFTO film of 0.5 u.c. (refs. ^8,16^). Note that in the Aurivillius system, such CDWs are known to be nucleated by substrate step edges^17^. Thus, despite their rigid epitaxial relationship, the BTO and BFTO in the bilayer adhere to their respective polar anisotropies. The domain configuration of the BTO–BFTO heterostructure, thus, resembles a partial flux closure (Fig. 2c). In contrast to spontaneously forming flux-closure domains (quadrant domains) within a single material, we achieve this closure by combining two materials with intrinsically different polar axes. In ferroic materials, flux-closure domain formation efficiently reduces the field energy by forming closed loops of field lines within the material^18^. Specifically, in ferroelectrics, flux closure^11,12^ effectively minimizes polarization discontinuities and bound-charge accumulation at the domain boundaries or interfaces. In our case, this means that the depolarizing field acting on BTO is successfully defeated, allowing the out-of-plane polarization to establish from the very first unit cell.

Remarkably, we observe that the polarization in BTO (5–10 nm) can be reversibly poled with a scanning probe tip (±5 V) (Fig. 2d,e). Volume-sensitive SHG imaging (Fig. 2f) confirms the ferroelectric nature of the switch and excludes surface-related phenomena as a possible origin of the PFM contrast (Fig. 2d). This is rather unexpected since ferroelectric poling ordinarily requires the insertion of a conducting bottom electrode. To understand why electric poling in the absence of a metallic bottom contact is possible, we investigate the BTO poling behaviour with BFTO films of different thicknesses. We find that a minimum thickness of 1.5 u.c. (~6 nm) is required for a BTO polarization switch (Supplementary Fig. 4). Tomographic PFM^19^ on a 5-u.c. BFTO film reveals that at this depth, the density of CDWs increases. Tomographic reconstructions with views from both above (Fig. 2g) and below (Fig. 2h) show that the in-plane-polarized stripe-domain architecture at the bottom interface gradually turns into a random distribution of smaller domains towards the top interface. We conclude that the increased population of CDWs, and the corresponding density of mobile charges in the buffer layers, emulates the functionality of a counterelectrode (Fig. 2i). Note that the threshold of 1.5 u.c. may be further reduced by selecting alternative Aurivillius compounds with fewer perovskite subunits. The CDW density may also be increased through the controlled formation of substrate step terraces using higher miscut angles or thermal annealing^17^. Supplementary Note 3 provides additional technical details related to the electrode-free poling of the top BTO layer.

### Using in-plane-polarized buffer for domain engineering

So far, we considered an inherently out-of-plane-polarized ferroelectric BTO. Replacing BTO with a ferroelectric that has its own in-plane polarization components could bring additional degrees of freedom to the ferroelectric domain and domain-wall engineering in our flux-closing system. To explore this, we grow (001)_pc_-oriented BFO—the only known robust room-temperature multiferroic—onto the BFTO buffer. Its eight possible polarization directions along the pseudocubic 〈111〉_pc_ axis^20^ lead to a rich variety of possible ferroelectric domain and domain-wall configurations with out-of-plane as well as in-plane polarization components.

**Fig. 3:**
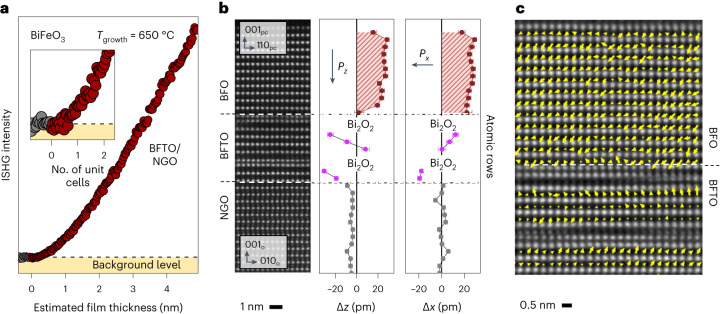
Polarization continuity at the BFO–BFTO interface. **a**, ISHG signal tracking of the out-of-plane polarization in the BFO thin film during its deposition on 1 u.c. of BFTO (red symbols). The grey circles correspond to the background ISHG signal prior to the BFO deposition. **b**, HAADF-STEM image of the coherently strained BFO–BFTO–NGO heterostructure. We note that the charged-surface termination of NGO causes an initial growth of BFTO with less than four perovskite sublayers. Out-of-plane (Δ*z*) and in-plane (Δ*x*) polar displacements of the B-site atomic columns from the centre of their two nearest A-site neighbours extracted from the HAADF-STEM image confirm the presence of BFO polarization from the first unit cell. The ferroelectric polarization is directed opposite to these displacements and the error bars signify the standard error of the mean. **c**, Ferroelectric dipole map at the BFO–BFTO interface overlaid with the HAADF-STEM image.

We monitor the ISHG signal during BFO deposition onto a BFTO layer of a single unit cell. As in the case of BTO, the ISHG yield (Fig. 3a) shows an onset of ferroelectricity in the BFO film with the first unit cell, thus demonstrating the material-independent potential of our flux-closure-like architecture in defeating depolarizing fields. Post-deposition STEM imaging (Fig. 3b) reveals the high crystalline quality of our BFO (12 u.c.)–BFTO (1 u.c.)–NGO heterostructure with no dead layer at the BFO–BFTO interface, as highlighted by the averaged dipolar displacements extracted from this image. The atomically resolved electric-dipole mapping of this interface is displayed in Fig. 3c.

To uncover how the interfacial flux closure affects the domain pattern and the domain-wall architecture in a BFO film of 25 nm, we opt for high-resolution scanning probe measurements for which a fully strained conducting LSMO layer is inserted underneath the flux-closing BFO–BFTO heterostructure. The charge screening provided by LSMO prevents the formation of domains in the Aurivillius buffer (Supplementary Note 4), which simplifies the PFM investigations as any LPFM contrast can now be attributed to domain formation in the BFO layer. Note that the insertion of LSMO maintains the respective polar anisotropies in the BFO–BFTO bilayer and hence its flux-closing properties, as deduced from PFM measurements (Supplementary Notes 4 and 5).

**Fig. 4:**
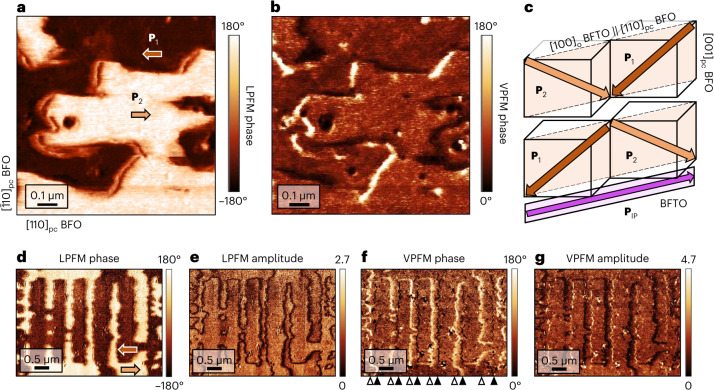
Polar homochiral Néel domain walls in BFO grown on BFTO. **a**, LPFM phase scan reveals [$11\bar{1}$]_pc_ (**P**_2_) and [$\bar{1}\bar{1}\bar{1}$]_pc_ (**P**_1_) as the only two in-plane polarization components in BFO when deposited on BFTO. **b**, VPFM phase scan shows a downward polarization across the film with only scattered local out-of-plane-polarized features. The associated PFM amplitude signals for **a** and **b** are shown in Supplementary Fig. 14. **c**, Schematic of the in-plane 109° HH and TT CDWs stabilized by the in-plane-polarized buffer. **d**,**e**, LPFM phase (**d**) and amplitude (**e**) scans show uniaxial in-plane-polarized BFO domains nucleated using the trailing field of a scanning probe tip. **f**, VPFM phase scan shows the out-of-plane polarization at the artificially created BFO domain walls (Supplementary Note 6 shows additional verification). Polarization rotation at the walls is identical to that of pristine domain walls (**b**): each HH wall exhibits a downward polarized state (black markers), whereas each TT wall is associated with an upward polarization (white markers), suggesting a uniform domain-wall chirality, as depicted in Fig. 5a. **g**, The VPFM amplitude increases at the HH walls and decreases at the TT walls. This reflects that the bottom BFTO layer favours a downward-oriented polarization and suppresses an upward-oriented polarization in the BFO layer.

The two LPFM brightness levels (Fig. 4a) suggest two in-plane polarization components in the BFO film. As shown in Supplementary Note 5, these are parallel to the uniaxial polarization axis of the BFTO. The VPFM phase signal is largely uniform (Fig. 4b), aside from some linear and dot-like discontinuities, which will be discussed later. This is consistent with a downward polarization across the BFO film. Note that this out-of-plane polarization orientation is opposite to the one stabilized in the BTO films on BFTO and is dictated by both charge accumulation at the interface^3,21^ and by the similarity between the chemical compositions of BFO and BFTO. This leads to initial homoepitaxial-like growth and electric dipoles pointing towards the fluorite-like BFTO planes^22^, as is generally known for Aurivillius compounds (Supplementary Note 1).

Out of the eight possible domain states, the pristine BFO in our heterostructure exhibits only two states, namely, those with a polarization pointing along [$11\bar{1}$]_pc_ or [$\bar{1}\bar{1}\bar{1}$]_pc_, denoted as **P**_1_ and **P**_2_, respectively (Fig. 4c). This geometry results in the formation of in-plane 109° domain walls, in which—unlike in commonly observed 109° domain walls in BFO films—the out-of-plane polarization component remains downward oriented from one domain to the other (Fig. 4c). Such a polarization configuration has never been observed in BFO films and shows that the in-plane-polarized buffer is a powerful tool for domain engineering.

### Stabilization of polar homochiral domain walls in BFO

**Fig. 5:**
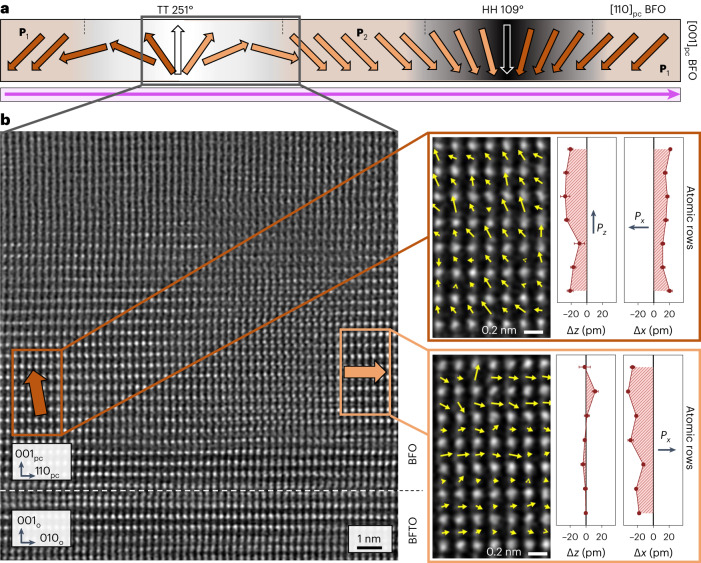
Polarization rotation at the non-Ising domain walls in the BFO film on BFTO–LSMO. **a**, Polarization rotation profiles in the (110)_pc_ plane across the HH and TT domain walls (deduced from Fig. 4d–g). The homochirality of the walls implies a net chirality in the BFO film, probably arising from the symmetry breaking of the in-plane-polarized BFTO buffer underneath. **b**, HAADF-STEM image showing the polarization rotation at the non-Ising TT domain wall in the BFO film on BFTO–LSMO. Atomically resolved imaging of BFO polar displacements in the selected areas (out of plane (Δ*z*) and in plane (Δ*x*)) confirms that the polarization rotates from **P**_1_ to **P**_2_ via an upward-oriented out-of-plane polarization. The ferroelectric polarization is directed opposite to these displacements and the error bars signify the standard error of the mean.

LPFM and VPFM images (Fig. 4a–b,d–g) reveal the in-plane and out-of-plane distributions of the BFO polarization associated with its domains and domain walls in both pristine state and after local electric-field poling using the scanning probe tip, respectively. To our surprise, the domain walls in the VPFM images appear as an alternating sequence of dark (downward polarized) and bright (upward polarized) lines. This implies a uniform sense of rotation of polarization across all of the walls or, in other words, the BFO domain walls are homochiral (Fig. 5a). The observed behaviour is drastically different from that of the classical out-of-plane 109° domain walls^23^, and was confirmed on six different samples. To exclude artifacts in the piezoresponse data as a trivial explanation for the observed domain-wall homochirality, we verified it with local conduction measurements using conductive atomic force microscopy and detailed PFM investigations (Supplementary Note 7). Most importantly, high-angle annular dark-field (HAADF) scanning transmission electron microscopy (STEM) imaging confirms the inferred polarization textures for the BFO domain walls in BFO–BFTO–LSMO (Fig. 5b) for a segment of one of the TT domain walls. Here the atomic displacements mapped for the areas marked with the coloured boxes highlight a rotation of polarization with an upward-oriented out-of-plane component at this TT wall, in line with the conclusions reached from PFM investigations.

The most striking aspect of this observation is that in the **P**_1_–**P**_2_ domain wall (Fig. 5a, left), the dipolar reorientation prefers a 251° ‘detour’ over the expected 109° rotation. To highlight the unusual nature of this state, we propose the notion of a 251° wall in this case. Note that the association of the HH walls to the 109° rotation and the TT walls to the 251° rotation leads to a non-zero net chirality of ferroelectric domain walls in our heterostructure, which is not exhibited by a BFO film deposited directly onto LSMO (Supplementary Note 8).

The homochirality of the BFO domain walls and the fact that a 251° wall is usually energetically more costly than a 109° wall show that the BFTO buffer exerts a pronounced symmetry-breaking effect. Its manifestation is reminiscent of the domain-wall homochirality observed in magnetically ordered heterostructures, where it is a consequence of the Dzyaloshinskii–Moriya interaction (DMI)^24,25^, arising from symmetry breaking at magnetic surfaces or interfaces. Here, in close analogy, an interface with an in-plane-polarized BFTO layer reduces symmetry and thus appears to give rise to the non-collinear polar textures that we observe at the BFO domain walls. Mirroring the free-energy invariant describing the DMI in chiral magnets and antiferromagnets^26–28^, the corresponding DMI-like^29^ invariant for polarization can be expressed as *E*_DM_ ∝ (*P*_*x*_∂_*x*_*P*_*z*_ − *P*_*z*_∂_*x*_*P*_*x*_), where *x* corresponds to the in-plane polarization axis and *z* points to the out-of-plane one. As for chiral magnets, it sets a deterministic sense of rotation of the order parameter across the domain walls, as we demonstrate in phase-field simulations (Supplementary Note 9). Such ferroelectric DMI-like interactions have so far mostly been discussed from a theoretical standpoint^29,30^, and the emergence of 251° domain walls in our BFO films is probably one of its first experimental observations. All this suggests the use of an in-plane-polarized buffer as an unforeseen route for the stabilization of polar homochirality^31,32^.

### Outlook

Our work introduces epitaxial flux-closing heterostructures that stabilize an out-of-plane polarization in ultrathin BTO and BFO right from the very first unit cell. The interfacial symmetry breaking, occurring between in-plane- and out-of-plane-polarized layers, gives rise to homochirality at the domain walls of the BFO film. In particular, it leads to 251° domain walls in our BFO films, a probable signature of a DMI-like behaviour in ferroelectrics. These results demonstrate that polar insulators can be more effective than metals in screening the detrimental depolarizing field in the ultrathin regime. Furthermore, the option to replace the metallic bottom electrodes with an in-plane-polarized buffer may facilitate the downsizing of device architectures. Both aspects could make the epitaxial combination of perpendicular polar anisotropies a powerful tool in oxide-electronics research.

## Methods

### Heterostructure growth

The thin films and heterostructures were grown on NGO (001)_o_ substrates by pulsed laser deposition, using a KrF excimer laser at 248 nm. The laser fluence, repetition rate, substrate temperature and growth pressure set for individual layers are as follows: BFTO: 0.9 J cm^−2^, 2 Hz, 650 °C, 0.075 mbar O_2_; BTO: 0.9 J cm^−2^, 2 Hz, 650 °C, 0.015 mbar O_2_; BFO: 0.7 J cm^−2^, 8 Hz, 630 °C, 0.125 mbar O_2_; LSMO: 0.9 J cm^−2^, 1 Hz, 650 °C, 0.035 mbar O_2_. The thickness of the thin films was monitored using a combination of reflection high-energy electron diffraction during growth and X-ray reflectivity ex situ.

### Optical SHG

ISHG was probed in reflection in the pulsed laser deposition growth chamber^3^. The probe beam of 860 nm (BFO) and 1,200 nm (BTO) was incident onto the sample with a pulse energy of 20 μJ and onto a spot size of 250 μm in diameter. The ISHG signal was detected using a monochromator and a photomultiplier system^3^. ISHG plots display an averaged intensity for every two (Fig. 1c) or three (Fig. 3a) data points measured.

An ex situ reflection setup with a 45° angle of incidence was used to obtain a non-zero SHG signal from the BTO out-of-plane polarization and to spatially resolve the SHG images of poled BTO–BFTO with a pulse energy of 25 μJ. The generated SHG light was collected with a ×20 long-working-distance microscope objective and projected onto a liquid-nitrogen-cooled back-illuminated chip of a charge-coupled device camera (Symphony II, Horiba Jobin Yvon). To gain domain contrast, an interference technique is employed. Here we took advantage of the SHG light produced by the surface of the BTO film during SHG imaging performed in reflection. This light does not couple to the ferroelectric order and hence is not affected by the polarization direction. The superposition of the SHG contributions emitted from oppositely polarized areas and surface contribution leads to constructive and destructive interference^33^ and results in a pronounced difference in the SHG yield between the upward- and downward-polarized regions. To account for the 45° reflection geometry, a trapezoidal perspective correction with the following transformation matrix *T* was applied to all the images with the GIMP software (v. 2.10.24) to revert the poled region into a square shape.

$$T=\left(\begin{array}{lll}1.2405&-0.0382&-21.6563\\ -0.0764&1.0419&1.8580\\ -0.0004&0.0003&1.0000\end{array}\right)$$

### PFM

The scanning probe microscopy measurements were recorded using an NT-MDT NTEGRA scanning probe microscope and two external SR830 lock-in detectors (Stanford Research) for the simultaneous acquisition of in-plane and out-of-plane piezoresponse. Data acquisition was performed using a 2.3 V a.c. modulation at 70 kHz applied to the Pt-coated tip (HQ:NSC35/Pt). Sharp diamond probes (AD-2.8-SS) were used for the high-resolution PFM images. Deflection and torsion modes were recorded when measuring with the cantilever perpendicular to the uniaxial polarization axis of BFTO/BFO. The LPFM and VPFM images were simultaneously recorded in Cartesian coordinates (using *X* and *Y* outputs of the external lock-in amplifiers) after minimizing the background piezoresponse and calibrating the polarization directions on periodically poled LiNbO_3_ (ref. ^34^). The collected data are then transformed into polar coordinates (*R* and *θ*) using the following equations: *X* = *R* cos(*θ* + *ϕ*) + *B*_*X*_ and *Y* = *R* sin(*θ* + *ϕ*) + *B*_*Y*_. The phase offset *ϕ* and components of the background piezoresponse *B*_*X*_ and *B*_*Y*_ along *X* and *Y*, respectively, are manually adjusted.

PFM phase images are plotted with a phase variation from −180° to 180°, the only exception being the VPFM images of homochiral BFO domain walls, as the scale from 0° to 180° helps to visualize the corresponding phase changes at the domain walls in the uniformly downward-polarized BFO film.

### PFM tomography

Tomographic atomic force microscopy (AFM) is based on sequential AFM imaging and probe-based nanomechanical sample milling^35^. Operating in the PFM mode^19^, specifically when biasing the conducting tip with an a.c. field oscillating at the torsional contact resonance, volumetrically mapping the in-plane domain contrast is possible. A PFM tomogram constructed from a sequence of 95 images, all in the same field of view but for gradually diminishing film thicknesses, is presented in Fig. 2g,h, which overall comprises more than one million 15.7 × 15.7 × 1.0 nm^3^ voxels of local piezoresponse signals. The colour contrast depicts the product of the measured amplitude and the sign of the phase for in-plane torsion of the cantilever, which is the lateral piezoresponse. The AFM equipment (Asylum Research Cypher VRS) is operated in the contact mode using doped diamond probes (AppNano DD-ACTA). A setpoint of approximately 2.24 μN is sufficient to mill this specimen, whereas torsional PFM imaging is performed with a 1 V peak-to-peak a.c. bias at a frequency of approximately 2.8 MHz. The amplitude and phase of the locally vibrating lever are simultaneously acquired via a lock-in amplifier (Zurich Instruments MFLI).

### STEM

Cross-sectional specimens were prepared by focused-ion-beam milling with an FEI Helios NanoLab 660 G3 UC instrument. HAADF-STEM imaging was attained with an FEI Titan Themis microscope equipped with a spherical-aberration probe corrector (CEOS DCOR) operated at 300 kV. A probe semi-convergence angle of 18 mrad was set in combination with an annular semi-detection range of the HAADF detector of 66–200 mrad. The atomic column positions in the HAADF-STEM images were fitted with picometre precision^36,37^. In Figs. 3b and 5b, the Δ*z* and Δ*x* polar displacements of the B-site atomic columns in the image plane from the centre of their two nearest A-site neighbours are along the [100]_pc_ and [011]_pc_ directions, respectively.

## Online content

Any methods, additional references, Nature Portfolio reporting summaries, source data, extended data, supplementary information, acknowledgements, peer review information; details of author contributions and competing interests; and statements of data and code availability are available at 10.1038/s41563-023-01674-2.

### Supplementary information

Supplementary Information Supplementary Notes 1–9, Figs. 1–21 and Tables 1 and 2.

## Data Availability

The dataset that supports the findings of this study is available via the ETH Zurich Research Collection at 10.3929/ethz-b-000626823.
