# Supplementary material for: Defeating depolarizing fields with artificial flux closure in ultrathin ferroelectrics
Source: Nat Mater. 2023 Oct 2;22(12):1492–8. doi: 10.1038/s41563-023-01674-2 (PMC10713449; doi:10.1038/s41563-023-01674-2)
Supplement: Supplementary file 1 — Supplementary Notes 1–9, Figs. 1–21 and Tables 1 and 2. [file 41563_2023_1674_MOESM1_ESM.pdf]

nature materials

Article https://doi.org/10.1038/s41563-023-01674-2

Defeating depolarizing fields with artificial flux closure in ultrathin ferroelectrics

In the format provided by the authors and unedited

Contents

Polarization configuration at the interfaces of flux-closing heterostructures (BTO|BFTO and BFO|BFTO) (incl. Supplementary Fig. 1) **3**

Epitaxial matching between perovskite oxides (BTO, BFO), Aurivillius compound BFTO and orthorhombic NGO $(001)_o$ substrate (incl. Supplementary Table 1 and Supplementary Fig. 2) **7**

In-plane-polarized BFTO buffer layer used as a counter-electrode for the BTO polarization switching (incl. Supplementary Fig. 3-7) **9**

Use of metallic LSMO buffer to alter the domain configurations in the BTO|BFTO and BFO|BFTO heterostructures (incl. Supplementary Fig. 8-12) **14**

Uniaxial in-plane polarization in BFO films grown on BFTO (1 u.c.)|LSMO (20 u.c.)|NGO $(001)_o$ (incl. Supplementary Fig. 13) **19**

Investigation of the domain-wall homochirality in BFO films on BFTO|LSMO (incl. Supplementary Fig. 14-16) **20**

Excluding the possibility of artifacts in the PFM measurements of the homochiral BFO domain walls (incl. Supplementary Fig. 17-19) **24**

Excluding epitaxial strain as the sole origin for the homochiral BFO domain walls (incl. Supplementary Fig. 20) **28**

Phase field simulation of the homochiral BFO domain walls (incl. Supplementary Table 2 and Supplementary Fig. 21) **29**

References **31**

Supplementary Note 1. Polarization configuration at the interfaces of flux-closing heterostructures (BTO|BFTO and BFO|BFTO) (incl. Supplementary Fig. 1)

To explain the orientation of the out-of-plane polarization in the perovskite BTO and BFO layers deposited on the in-plane-polarized BFTO, we now consider the charge configuration at the BTO|BFTO and BFO|BFTO interfaces. The BFTO crystalline lattice consists of a succession of charged atomic planes as depicted in Supplementary Fig. 1. The arising dipole moment is commonly referred to as layer polarization[1] and is instrumental in stabilizing and directing the out-of-plane polarization in the perovskite layers deposited on top of it. For the two interfaces described here, we can consider two possible BFTO surface terminations, either Bi^{3+} or O^{2-}, inducing either a positive or negative charged interface, respectively (see Supplementary Fig. 1a and b). This surface charge dictates the preferred polarization direction for the perovskite layers grown on top of it.

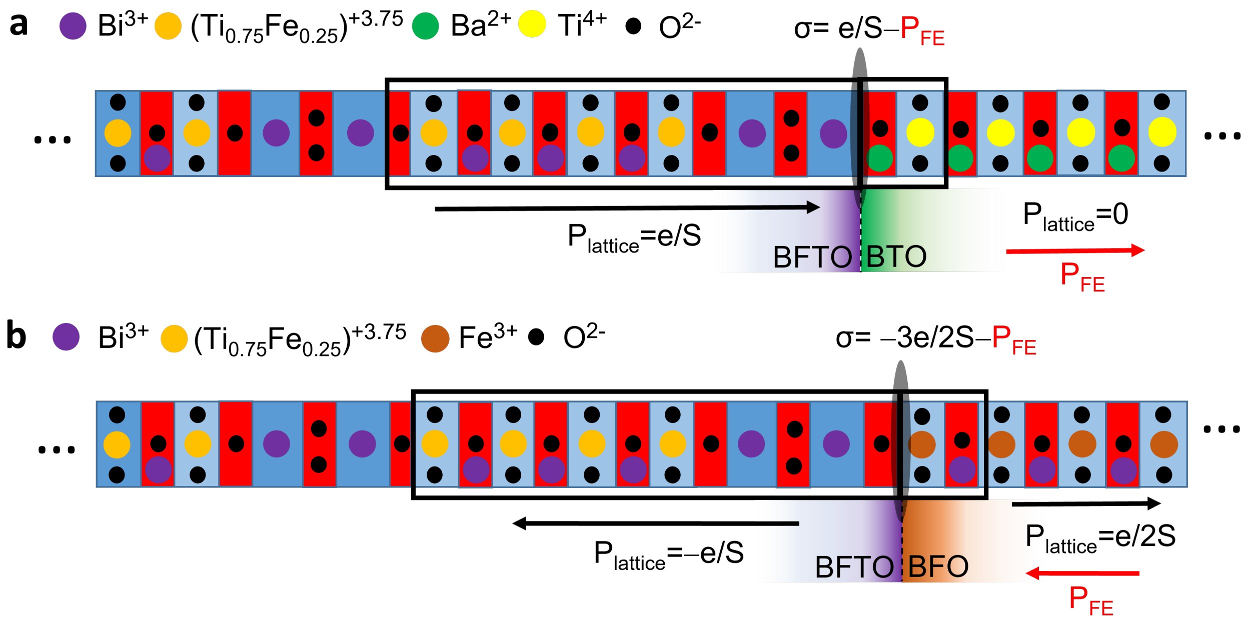

Supplementary Fig. 1. | Calculation of the bound charge at the BTO|BFTO (a) and BFO|BFTO (b) interface. Black boxes show the choices of unit cells used to calculate the discontinuity in the lattice polarization, black arrows indicate the lattice polarizations and red arrows indicate the preferred directions for the ferroelectric polarization. σ is the surface charge at the interface.

Upward polarization in BTO on BFTO. When BTO is grown on BFTO, the scenario shown in Supplementary Fig. 1a is realized, and BFTO films self-terminate on the $[Bi_2O_2]^{2+}$ fluorite-like layers[2]. Because of the different chemical compositions of the BTO and BFTO unit cells, we can identify a well-defined interface between the two compounds. We consider a net surface charge of the $[Bi_2O_2]^{2+}$ plane, which can be subdivided into two Bi^{3+} layers and a double-oxygen layer carrying an intermediate ionic charge of $4-$ in between them. The Bi^{3+} surface plane creates an accumulation of positive bound charges at the interface, see Supplementary Fig. 1a. These non-screened charges induce a polarization in the perovskite layer that counteracts the depolarizing field for the upward-polarized BTO that has neutral planes itself.

Downward polarization in BFO on BFTO. In the case of the BFO|BFTO interface, the similarity of the chemical composition of the two compounds (common Bi- and Fe-rich planes) allows for a homoepitaxial-like continuation of the structure at the interface, via a termination at the O^{2-} layer, as shown in Supplementary Fig. 1b. This leads to electric dipoles pointing towards the fluorite-like plane, as is generally known for Aurivillius compounds[3]. In the Aurivillius unit cell, the polarization in the perovskite blocks is directed toward the double-oxygen layer carrying an ionic charge of $4-$ within the $[Bi_2O_2]^{2+}$sheets[1]. While for a complete Aurivillius unit cell, consisting of four perovskite blocks sandwiched between two $[Bi_2O_2]^{2+}$ layers, the electric dipole ordering leads to a suppression of the net out-of-plane direction and a fully in-plane ferroelectric behavior, here it results in a distinct preference for the downward polarization in BFO[1]. In addition, in contrast to BTO, BFO possesses its own charged planes ($(FeO_2)^{1-}$ and $(BiO)^{1+}$), and the plane sequence in BFO films starting with the FeO_2 plane on BFTO, see Supplementary Fig. 1b, further supports a downward oriented ferroelectric polarization[4].

Calculations of interfacial bound charge. In order to describe the electrostatic boundary conditions at our grown interfaces between BFTO and BTO/BFO more rigorously, we calculate the bound charge arising at the interface for both perovskite compounds. The lattice polarization P_{lattice}

of the centrosymmetric lattice is calculated as:

$$P_{lattice} = \frac{1}{\Omega} \sum_i c_i Z_i \quad , \tag{1}$$

where c_i is the c-axis coordinate of each layer, Z_i is the corresponding formal charge and Ω is the unit-cell volume. In a bulk material, this sum is calculated within periodic boundary conditions which gives rise to an ill-defined position operator and a multivalued solution for P_{lattice}. As described by the modern theory of polarization, these values correspond to the different branches of the polarization lattice (see for example ref.[5]). For BFTO and BTO we obtain a zero-centered polarization lattice of $P_{\text{lattice}} = 0 \pm ne/S$, where n is an integer, while BFO has an off-centered polarization lattice of $P_{\text{lattice}} = e/2S \pm ne/S$, where S is the surface area of the unit cell perpendicular to the layer.

While for the ferroelectric properties of a bulk material the lattice polarization can be neglected, in the presence of an interface between materials with different lattice polarizations this property becomes highly relevant, as changes in the lattice polarization lead to the formation of bound charge at the interface. We calculate the bound charge for our heterostructures, for the surface configurations shown in Supplementary Fig. 1. We make use of the interface theorem of the modern theory of polarization[6], to ensure the same choice of the same polarization branch in both materials, using the condition of interface neutrality (absence of free charge or missing atoms at the interface)[7]. This allows us to calculate the bound charge at the interface, and we find $\sigma = e/S$ and $\sigma = -3e/2S$ for BFTO|BTO and BFTO|BFO interfaces, respectively.

These arising bound charges at the interface give rise to large electric fields in the materials and need to be screened in order to prevent divergence of the electric potential. For instance, this can be achieved by the accumulation of electrons/holes or by point defects. In the case of the interfaces between BFTO and out-of-plane-polarized BTO and BFO, the ferroelectricity of the top layer provides an intrinsic screening mechanism, as the appropriate orientation of the ferroelectric polarization reduces the discontinuity of the polar lattice. The bound charge, in this case, is given

by:

$$\rho_b = -\nabla \cdot (P_{\text{lattice}} + P_{\text{FE}}) \tag{2}$$

which means that the surface charge at the interface is:

$$\sigma_b = P^{\text{BFTO}}_{\text{lattice}} - P^{\text{FE}}_{\text{lattice}} - P^{\text{FE}}_{\text{FE}} = \Delta P_{\text{lattice}} - P_{\text{FE}} \tag{3}$$

Using the surface charges of $\sigma = e/S$ and $\sigma = -3e/2S$ for BFTO|BTO and BFTO|BFO, respectively, as calculated before, we can conclude that the interface introduces a strong preference for an up polarization in BTO and for a down polarization in BFO. Conversely, the bound charge induced by the interface compensates for the bound charge of the ferroelectric polarization, responsible for the depolarizing-field effects and critical thickness for ferroelectricity. As such, it can provide a way to stabilize a ferroelectric state even in ultrathin films, as we have shown in Fig. 1c and 3a of the main text.

Supplementary Note 2. Epitaxial matching between perovskite oxides (BTO, BFO), Aurivillius compound BFTO and orthorhombic NGO $(001)_o$ substrate (incl. Supplementary Table 1 and Supplementary Fig. 2)

Structural compatibility of BFO/BTO and BFTO. In this supplementary note, we describe the epitaxial relationship between the Aurivillius and perovskite phases on NGO $(001)_o$ substrate. These oxide phases are structurally compatible when their *c*-axes are parallel and point out of the film plane, while the perovskite unit cell is rotated in-plane by 45° with respect to the orthorhombic Aurivillius unit cell. This gives rise to compressively strained perovskite layers. The nominal epitaxial strain associated with each layer in the heterostructures is given in Supplementary Table 1.

	Lattice parameters (Å)			Mismatch (%)	
	a	b	$\sqrt{a^2+b^2}$	$[100]_{NGO}$	$[010]_{NGO}$
NGO[8]	5.428	5.498			
BTO[9]	4.000	4.000	5.657	−4.05	−2.81
BFO[10]	3.965	3.965	5.607	−3.20	−1.95
BFTO[11]	5.470	5.439		−0.21	+0.51

Supplementary Table 1. | Lattice parameters and the resulting lattice mismatch on NGO $(001)_o$ substrate. Epitaxy between 001-oriented pseudocubic unit cell (denoted pc) and orthorhombic unit cell (denoted o) is achieved when a_{pc} and b_{pc} are kept equal, while diagonals of the unit cell are matched to a_o and b_o[12]. This leads to the epitaxial relationship with $[100]_{pc} \parallel [1\bar{1}0]_o$ and $[010]_{pc} \parallel [110]_o$.

Ruling out a strain-induced reduction in critical thickness for ferroelectricity. Next, we investigate the effect of epitaxial strain on different substrates on the onset of polarization emergence in the ultrathin BTO. In Fig. 1c,d of the main text we saw that an in-plane-polarized insulating BFTO buffer layer on NGO $(001)_o$ can surpass the metallic LSMO electrode on STO (001) in stabilizing an out-of-plane-polarized ferroelectric state in the ultrathin BTO film. As compressive strain is usually associated with a higher spontaneous polarization and ferroelectric T_C[13,14] and could additionally decrease the critical thickness, this trend for polarization emergence on the two different substrates could be mistaken for a strain effect. This is because the NGO substrate exerts a nominally higher compressive strain (see Supplementary Table 1) on BTO than STO. Hence, we use

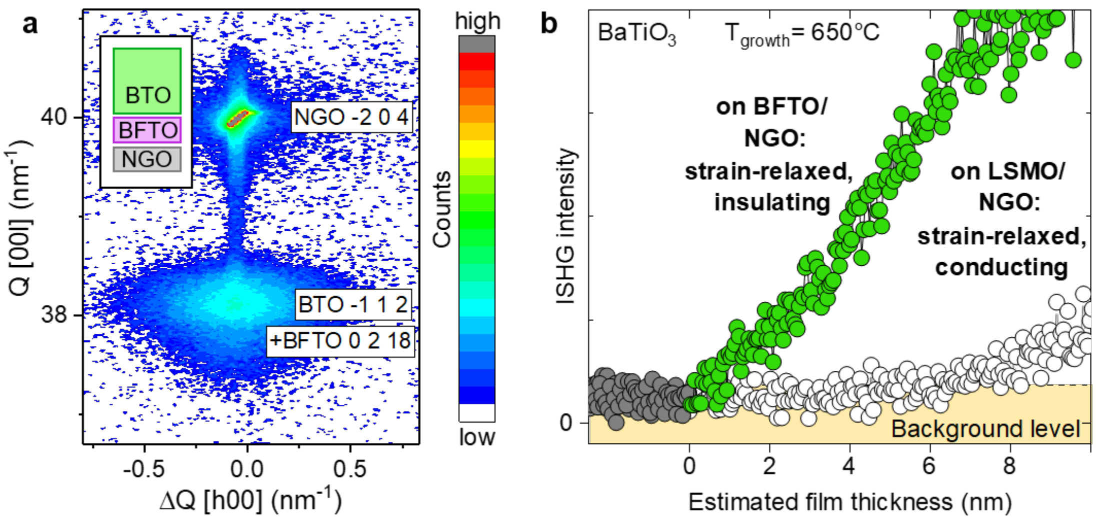

Supplementary Fig. 2. | Influence of strain relaxation on the critical thickness of BTO. **a**, Reciprocal space map (out-of-plane Q [00l] and in-plane ΔQ[h00]) around the NGO (−2 0 4) reflection reveal a partial BTO relaxation in the BTO|BFTO|NGO heterostructure sketched as inset. **b**, The ISHG signal tracking the BTO growth on an NGO $(001)_o$ substrate buffered with metallic LSMO (white) and with insulating in-plane-polarized BFTO (green). While both films are in the same strain state (partially relaxed), the zero critical thickness for the BTO ferroelectricity is only observed for the film grown on BFTO. This suggests that epitaxial strain cannot explain the observed zero critical thickness on BFTO so it can only be related to the polarization continuity at the BTO|BFTO interface.

reciprocal space mapping to investigate the strain state in the heterostructures. While BTO is fully strained on STO[15], the BTO layer is partially relaxed on NGO, see Supplementary Fig. 2a. This means that the less compressed BTO film shows an earlier onset of polarization, suggesting that the strain-related effects cannot explain the zero critical thickness for the polarization of BTO on the BFTO buffer. In Supplementary Fig. 2b we show the comparison of the ISHG signals collected during the BTO growth on the same type of NGO substrate, but on two different buffer materials: metallic LSMO and insulating, in-plane-polarized BFTO. Here, in the identical strain state, the BFTO buffer supports the emergence of polarization in BTO significantly better than the metallic LSMO, despite the charge screening the metal provides. All of this allows us to exclude epitaxial strain as the origin for zero critical thickness in BTO films grown on BFTO.

Supplementary Note 3. In-plane-polarized BFTO buffer layer used as a counter-electrode for the BTO polarization switching (incl. Supplementary Fig. 3-7)

Probing buried BFTO domains in BTO|BFTO. In this supplementary note, we investigate how the domain configuration in the BFTO buffer influences the switching properties of the top BTO layer. The LPFM images characteristic of the ultrathin BFTO buffer layer can be measured through the BTO layer deposited on top of it. The contrast between 180° stripe domains is recorded in the LPFM phase (Supplementary Fig. 3a), and here the minimum LPFM amplitude is observed at the walls (Supplementary Fig. 3b). Such in-plane-polarized stripe domains are associated with the ultrathin layers of the Aurivillius phase[16,17], suggesting that the intrinsically preferred configuration of in-plane-polarized domains in the Aurivillius buffer is not affected by the integration into the BTO|BFTO heterostructure. It thus validates the model of artificial flux closure created by these two layers of perpendicular polar anisotropies, as schematized in Fig. 2c of the main text.

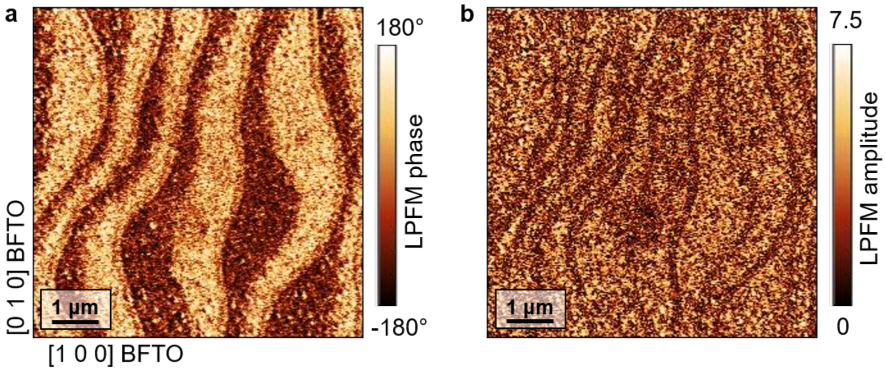

Supplementary Fig. 3. | LPFM images recorded for the BTO|BFTO heterostructure. LPFM (**a**) phase and (**b**) amplitude images of the uniaxial in-plane-polarized domains in our BTO|BFTO heterostructure, corresponding to Fig. 2b in the main text.

The BTO poling dependence on the BFTO thickness. In-plane-polarized BFTO buffer layers not only stabilize the BTO polarization but can also be used as bottom electrodes (in place of standard metallic buffer layers such as $SrRuO_3$) to facilitate the BTO polarization switching. The switching properties are dependent on the BFTO thickness. Thicker BFTO layers have a higher density of charged domain walls and the associated screening charges. We observe that 1.5 u.c.

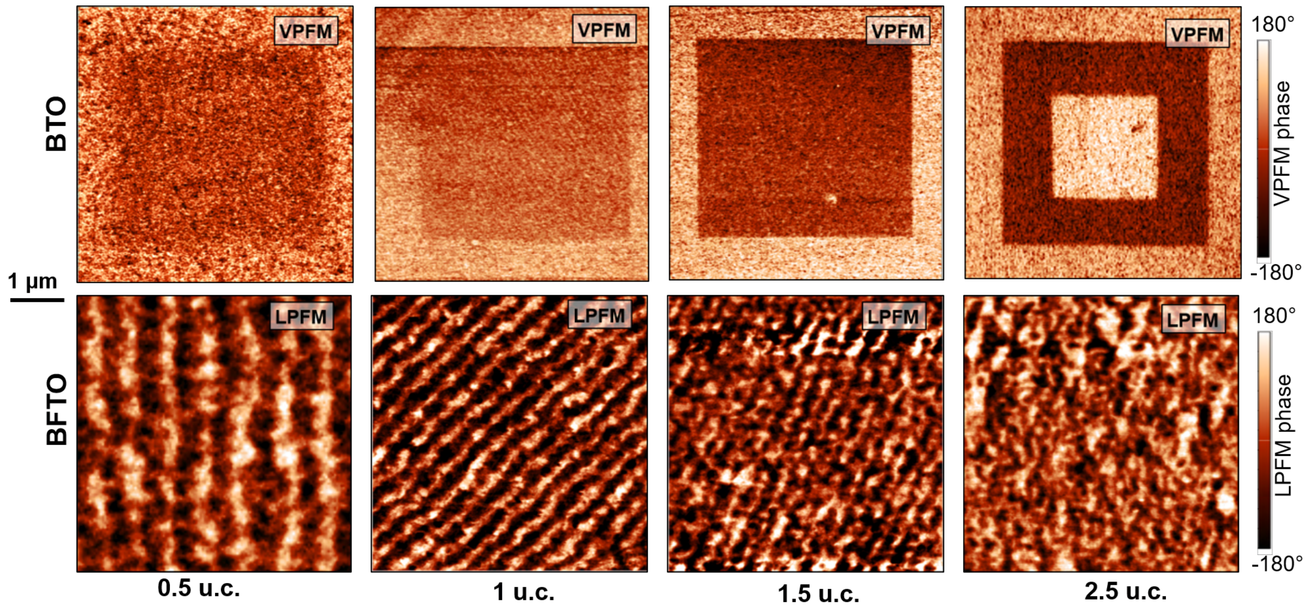

Supplementary Fig. 4. | BTO switchability as a function of the BFTO thickness and its in-plane domain configuration. PFM was performed on a series of BTO|BFTO heterostructures with a fixed BTO thickness of 10 nm and the BFTO thickness ranging from 0.5 to 2.5 u.c. VPFM phase scans (top row) show the contrast after poling the BTO films with ± 15 V. LPFM phase scans (bottom row) show the corresponding domain configurations in the in-plane-polarized BFTO buffer recorded on single-layer BFTO films. Metal-electrode-free poling is successful starting from the BFTO thickness of 1.5 u.c. ($\simeq 6$ nm) at which the BFTO domain pattern changes from stripes to smaller randomly arranged domains, suggesting that the increased domain-wall density is the key for the switchability of the BTO top layer.

thickness is the threshold BFTO thickness required for the switching, see Supplementary Fig. 4. This correlates with the BFTO thickness at which large in-plane stripe domains start splitting into smaller in-plane domains (see LPFM images in Supplementary Fig. 4), in this way increasing the density of conducting HH and TT domain walls in the buffer layer and thus facilitating polarization switching.

The existence of two different domain configurations in BFTO films, associated with different thickness regimes (below and above 1.5 u.c. $\simeq 6$ nm of BFTO), is further confirmed by AFM tomography performed on a 5-u.c.-thick ($\simeq 25$ nm) BFTO film. Supplementary Fig. 5 displays a montage of piezoresponse maps every 1 nm throughout the thickness of the tomographic AFM dataset. A gradual transition from well-ordered, aligned stripe domains near the substrate to a

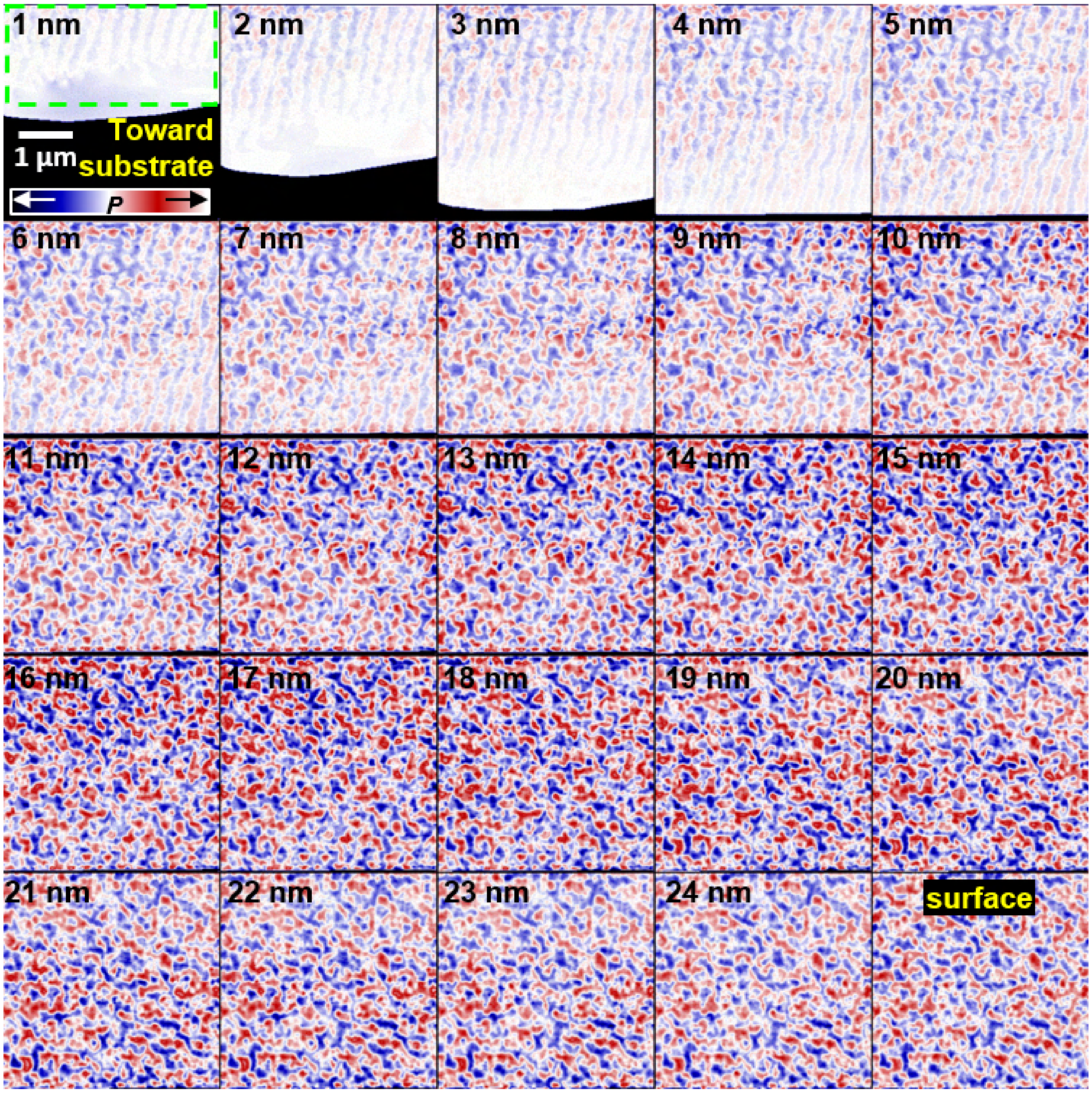

Supplementary Fig. 5. | **Piezoresponse maps (4 µm × 4 µm) collected throughout the thickness of a 5-u.c.-thick layer of BFTO.** The piezoresponse maps (LPFM phase) were extracted from the tomographic AFM dataset at every 1 nm of the BFTO film ($\simeq$ 25 nm). The tomograms are arranged from nearest the substrate (top left) toward the surface of the BFTO film (bottom right). The black regions in the first few frames represent areas that were not accessed for a given depth.

high density of columnar domains near the surface is apparent. Reproducibility was verified by acquiring multiple tomographs from the same specimen, with equivalent results.

Robustness of the BFTO domain configuration upon the BTO poling. Importantly, the BFTO domain configuration stays unchanged upon out-of-plane electric field application. Supplementary Fig. 6a shows the volume-sensitive SHG image of a poled region in BTO grown on BFTO|NGO. The corresponding line-scan profile of the SHG intensity across the pristine, the switched, and the back-switched regions is shown in Supplementary Fig. 6b. The identical SHG intensity levels in the pristine and poled back regions confirm the unchanged net polarization state of the buffer upon poling. Note that any deviation from the 180° stripe-domain architecture in the BFTO buffer layer during the BTO poling would lead to a non-zero SHG response, which would, in turn, alter the total SHG yield of the heterostructure. This suggests that the BTO polarization in the upper layer can be switched reversibly many times without perturbing the charged-domain configuration of the buffer BFTO layer beneath it. While the presence of charged domain walls is the key ingredient in the electrode-free poling of the top ferroelectric layer, they are not required for the stabilization of ferroelectricity in BTO from the first unit cell, see Supplementary Fig. 12.

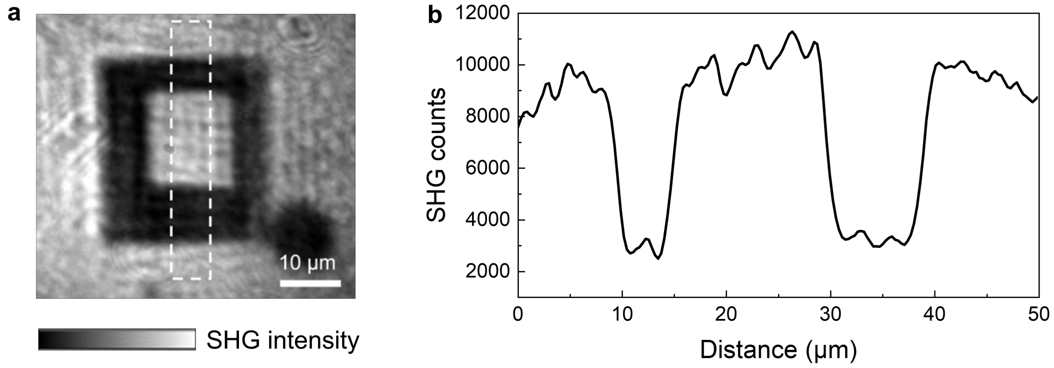

Supplementary Fig. 6. | **SHG imaging of the poled area in a BTO (10 nm)** | **BFTO (3.5 u.c.) heterostructure. a**, SHG imaging of a 30×30 μm^2 poled area confirms the ferroelectric nature of the switch. **b**, The average line profile of the area marked in **a** shows that SHG intensity associated with the pristine and reversibly switched area is the same, suggesting that the BFTO domain state is unchanged upon BTO switching.

Using PFM, the buried domain structure of the BFTO buffer was investigated following the top BTO poling. The surface sensitivity of the technique makes the measurement challenging. Thick BTO layers exhibit a strong VPFM signal but prevent the detection of a lateral piezoresponse signal from the buffer below. However, when the BTO films are thin enough to enable

the detection of the piezoresponse of the buried BFTO, they exhibit a weakened VPFM response, which complicates interpretation. For completeness we show the PFM images of the latter configuration confirming, within our experiment detection limits, the persistence of the BFTO domain architecture upon the local BTO poling, see Supplementary Fig. 7.

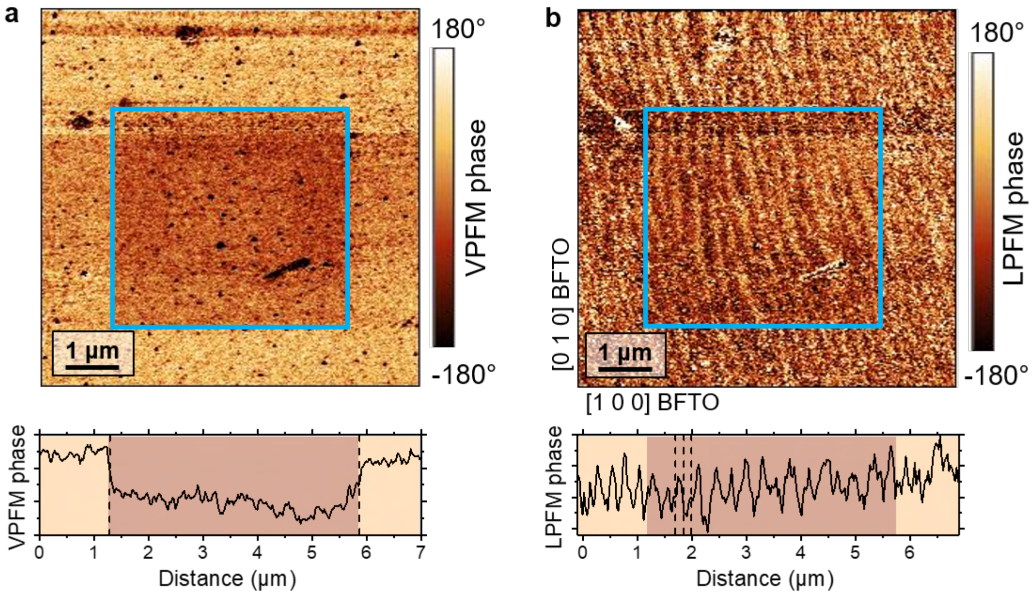

Supplementary Fig. 7. | PFM imaging of the poled area in a BTO (5 nm) | BFTO (1.5 u.c.) heterostructure. a, The VPFM phase scan shows a downward-poled area in BTO. **b**, The LPFM phase scan reveals stripe domains in the BFTO buffer layer underneath. The stripe-domain configuration remains unchanged in the poled area. Average horizontal line-scan profiles across the poled area (marked with a blue outline) are provided below the two PFM images. They confirm that the BFTO domain configuration remains unchanged after the poling of the BTO polarization.

Supplementary Note 4. Use of metallic LSMO buffer to alter the domain configurations in the BTO|BFTO and BFO|BFTO heterostructures (incl. Supplementary Fig. 8-12)

In this supplementary note, we demonstrate how the use of a metallic LSMO buffer triggers a single-domain configuration in the BFTO buffer layer and influences domain configurations of the flux-closing BTO|BFTO and BFO|BFTO heterostructures.

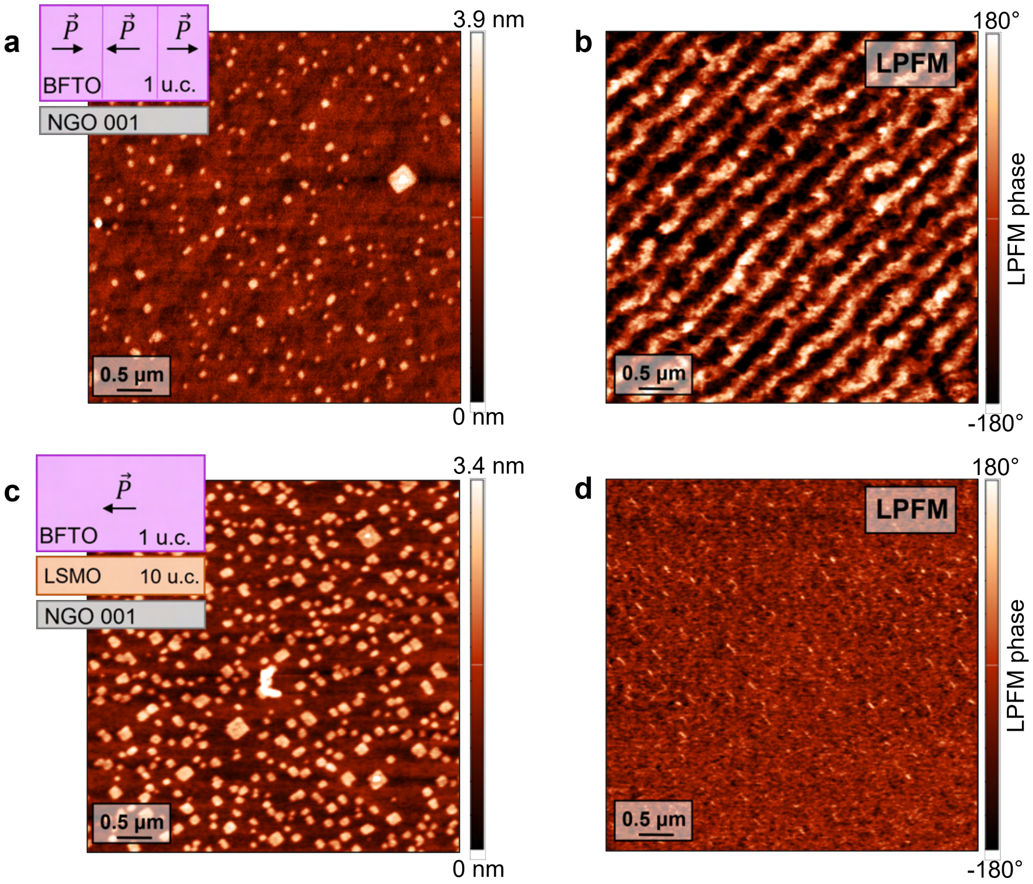

Supplementary Fig. 8. | Topography and LPFM scans recorded for a BFTO film (1 u.c.) deposited directly on NGO substrate and on a metallic LSMO electrode (10 u.c.). Topography (**a**, **c**) and LPFM phase images (**b**, **d**) recorded for 1-u.c.-thick BFTO layers grown directly on NGO substrate and on a 10-u.c.-thick metallic LSMO electrode, respectively. While stripe domains are clear when the film is grown on the substrate, no domains (only topography cross-talk) are resolved on the metallic electrode, suggesting that the BFTO film is in a single-domain configuration.

Single-domain state in BFTO films deposited on LSMO. We use a metallic LSMO buffer to suppress the formation of in-plane-polarized domains in the BFTO buffer to simplify our PFM

investigation of domain walls and domain configurations in the flux-closing heterostructures. 1-u.c.-thick BFTO film on LSMO shows no ferroelectric domain contrast in comparison to the stripe domains obtained for film growth directly on the NGO substrate, despite the same film thickness and morphology, see Supplementary Fig. 8. The uniform piezoresponse of the film points to a single-domain state.

In addition to PFM measurements, we performed ex-situ SHG studies to confirm the ferroelectricity in the films and unambiguously establish the orientation of the polarization axis. SHG polarimetry scans were performed by varying the polarization of the incoming light from 0° to 360° and detecting the outgoing light at $\alpha = 0°$ (a) and $\alpha = 90°$ (b) in reflection (Supplementary Fig. 9). They show a clear SHG fingerprint of the ordered state in the BFTO|LSMO|NGO heterostructures. The four-lobe and two-fold anisotropies of the emitted SHG light, respectively, are compatible with the *mm2* point group of the ferroelectric BFTO crystal structure[11] with a net polarization along the NGO *b*-axis, as demonstrated by the data fits. In combination with the absence of domain contrast in PFM measurements (Supplementary Fig. 8) and a negligible, surface-related, SHG signal detected for the metallic buffer layer alone (LSMO|NGO) (Supplementary Fig. 9), this allows us to unambiguously associate the SHG signal to a single-domain configuration in the BFTO film on LSMO, as opposed to sub-resolution nanodomains or paraelectric behavior.

Use of LSMO buffer for the BFO|BFTO heterostructure. The absence of domains in the BFTO layer simplifies the PFM investigations of the BFO|BFTO heterostructure as any LPFM contrast can now be attributed to in-plane-polarized domains in the BFO layer. Additionally, the BFO domains in the film grown on BFTO without the LSMO buffer layer are too small for a PFM analysis of the domain walls, see Supplementary Fig. 10. The insertion of a coherently strained LSMO (30 u.c.) beneath BFO|BFTO increases the size of the BFO domains and makes our investigations of its domain walls feasible and more convenient, see Fig. 4 in the main text. Note that there is no qualitative change to the overall domain state in the heterostructures with and without the LSMO buffer: the VPFM phase shows uniformly downward polarization with some discontinuities at the domain walls that are associated with their non-Ising character and the LPFM phase shows uniaxial

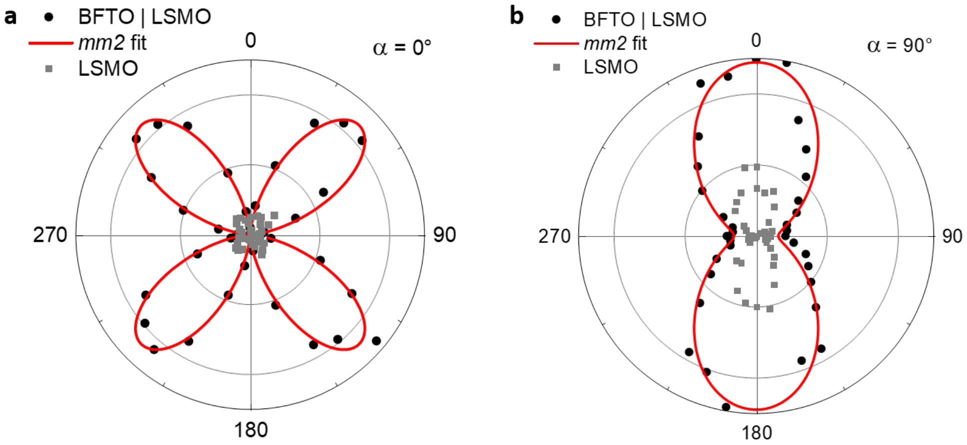

Supplementary Fig. 9. | Rotational SHG anisotropy recorded for the BFTO|LSMO bilayer and the LSMO metallic buffer alone. SHG polarimetry measurements were recorded in reflection where the polarization of the incoming light is varied from 0° to 360° and the outgoing light is detected at (**a**) $\alpha = 0°$ and (**b**) $\alpha = 90°$. The signal recorded for the BFTO|LSMO bilayer (black symbols) can be fit (red line) to the *mm2* point group symmetry of the ferroelectric BFTO crystal structure[11] with the net polarization along $[0 \pm 1\ 0]_{o\ NGO}$ using ♯SHAARP package[18]. Polarimetry measurements of the metallic LSMO electrode alone result in a significantly lower signal, further corroborating the ferroelectricity in the BFTO layer.

in-plane-polarized domains (along the *b*-axis of the NGO).

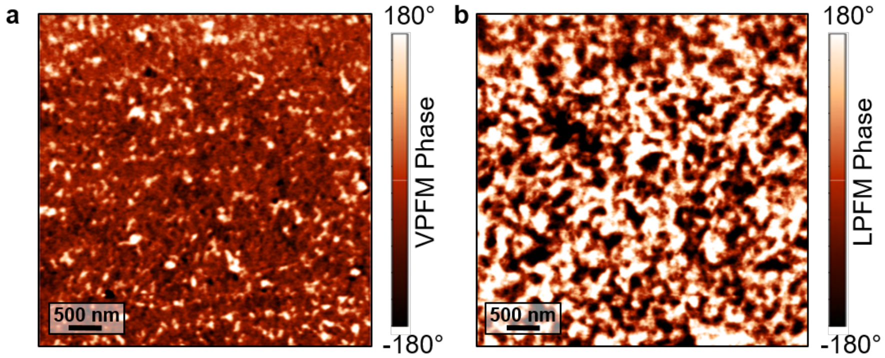

Supplementary Fig. 10. | VPFM and LPFM imaging of BFO domains on BFTO (1 u.c.) without LSMO. a, VPFM phase shows a downward polarization with some discontinuities at the domain walls. **b**, LPFM phase scan shows two in-plane-polarized domain states. While there is no qualitative change to the overall domain state compared to the BFO|BFTO|LSMO heterostructure, the size of BFO domains is significantly diminished, see Figure 4a,b for comparison. Therefore, the inserted LSMO layer not only simplifies the LPFM investigations but also makes the investigations of the BFO domain walls feasible.

Use of LSMO buffer for the BTO|BFTO heterostructure. As there is no in-plane oriented domain (*a*-domain) formation in BTO grown on BFTO (confirmed with XRD and PFM), we do not need the LSMO insertion for distinguishing the origin of in-plane-polarized domains we observe in PFM for BTO|BFTO heterostructures. Instead, the in-plane-polarized domains can be unambiguously associated with the BFTO layer, whereas VPFM contrast is associated with domains in the BTO top layer, see Figure 2 of the main manuscript. Nonetheless, the characterization of the BTO|BFTO bilayer with the LSMO buffer is shown in Supplementary Fig. 11. In contrast to the heterostructure with no metallic electrode, a single-domain configuration, enforced by the LSMO, is revealed in LPFM (Supplementary Fig. 11b). The uniform out-of-plane polarization of the BTO and its direction dictated by the BFTO charge planes remain unaffected (Supplementary Fig. 11a,c). We note that the BTO polarization switching (Supplementary Fig. 11c) in this heterostructure is enabled by the LSMO metallicity despite the absence of charged domain walls in the BFTO layer.

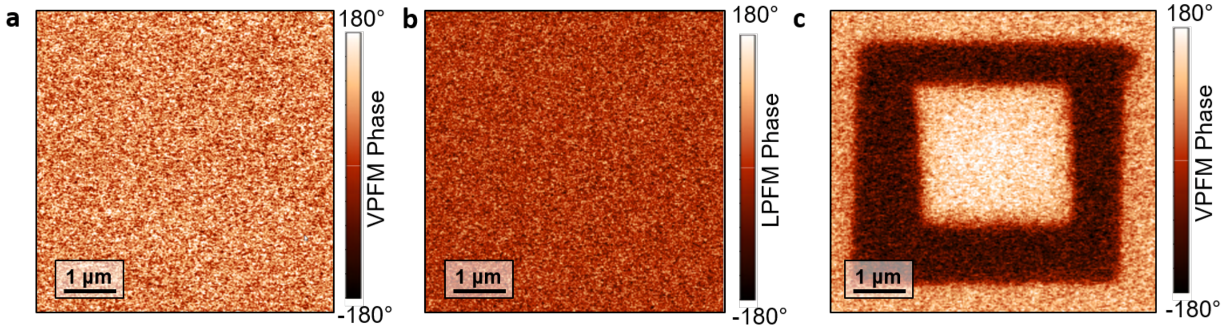

Supplementary Fig. 11. | PFM investigation of the BTO|BFTO heterostructure buffered with an LSMO electrode. a, VPFM phase shows a uniform upward polarization. **b**, LPFM phase shows no in-plane domains consistent with a BFTO layer in a single-domain configuration. **c**, VPFM phase contrast confirms the successful out-of-plane polarization reversal, similar to the reversible poling observed in the BTO|BFTO bilayer without the LSMO back-electrode.

Lastly, we investigate how the LSMO insertion affects the stabilization of the BTO polarization in the ultrathin regime. Supplementary Fig. 12 shows that in the BTO|BFTO heterostructure the BTO polarization emerges from the very first unit cell deposited, regardless of whether it is buffered with LSMO or not. This shows that polarization continuity at the BTO|BFTO interface is crucial for polarization stabilization, while charged domain walls in the BFTO buffer are not

(BFTO has no domain walls when on LSMO).

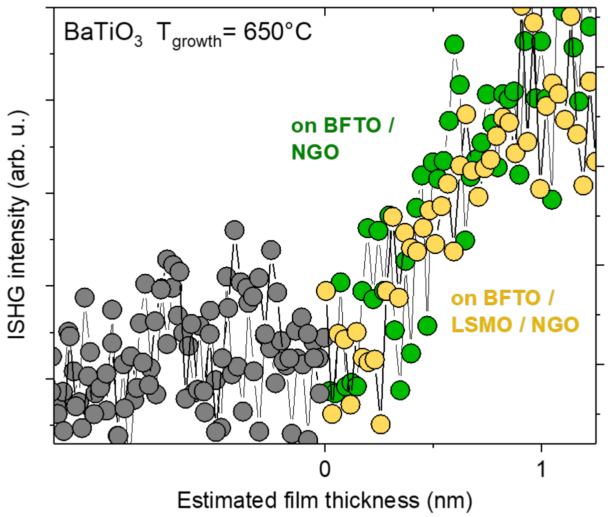

Supplementary Fig. 12. | Stabilization of the BTO polarization in flux-closing BTO|BFTO heterostructures with and without the LSMO back-electrode. ISHG signal tracking the BTO polarization during the early stage of its deposition on the BFTO buffer alone (green symbols) and the BFTO layer buffered with metallic LSMO (yellow symbols). The emergence of the BTO polarization is observed from the very beginning of its deposition in both heterostructures.

Supplementary Note 5. Uniaxial in-plane polarization in BFO films grown on BFTO (1 u.c.)|LSMO (20 u.c.)|NGO $(001)_o$ (incl. Supplementary Fig. 13)

In this supplementary note, we verify the uniaxial nature of in-plane polarization in our BFO films using vector LPFM measurements in three different sample orientations with respect to the cantilever, as shown in Supplementary Fig. 13. In-plane-polarized BFO domains only appear in the LPFM channel when the cantilever is aligned with the $[100]_o$ NGO axis, suggesting that the in-plane polarization lies along $[010]_o$ only.

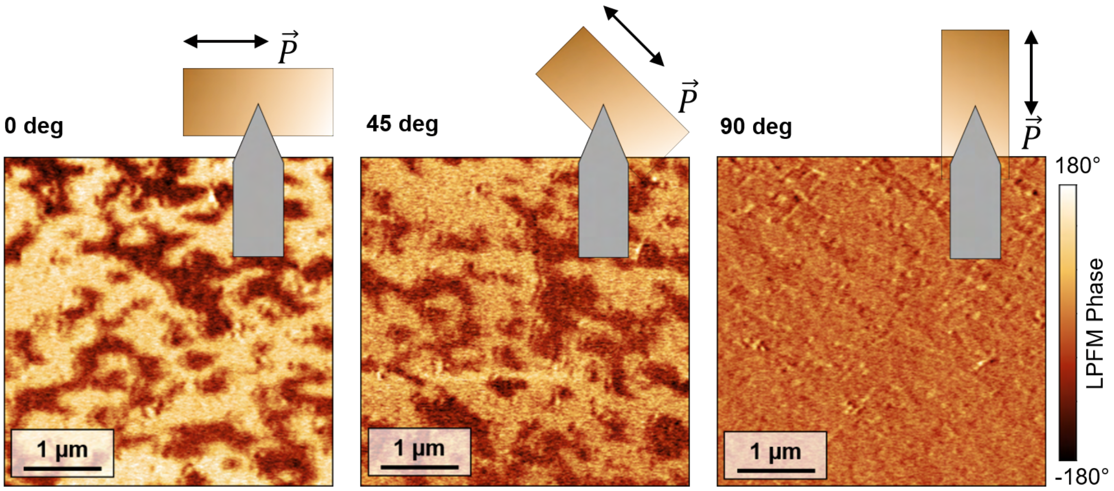

Supplementary Fig. 13. | Uniaxial in-plane polarization in BFO determined with vector PFM study. The uniaxial nature of in-plane polarization in BFO films on BFTO|LSMO|NGO was confirmed using vector PFM by separating the torsion and buckling modes of the cantilever. With the cantilever aligned with the $[100]_o$ NGO axis, the uniaxial polarization of BFO and BFTO beneath are at right angles to the cantilever, meaning that the piezoresponse is now predominantly recorded as the LPFM signal due to the torsion modes. At 45° sample rotation the domains are measured in both LPFM (torsion) and VPFM (buckling) channels, hence the LPFM signal weakens. With the sample rotated by 90° and the cantilever aligned with the $[010]_o$ NGO axis (a), the signal is now no longer in the LPFM channel and is instead recorded in the VPFM channel (not shown) as the buckling of the cantilever.

Supplementary Note 6. Investigation of the domain-wall homochirality in BFO films on BFTO|LSMO (incl. Supplementary Fig. 14-16)

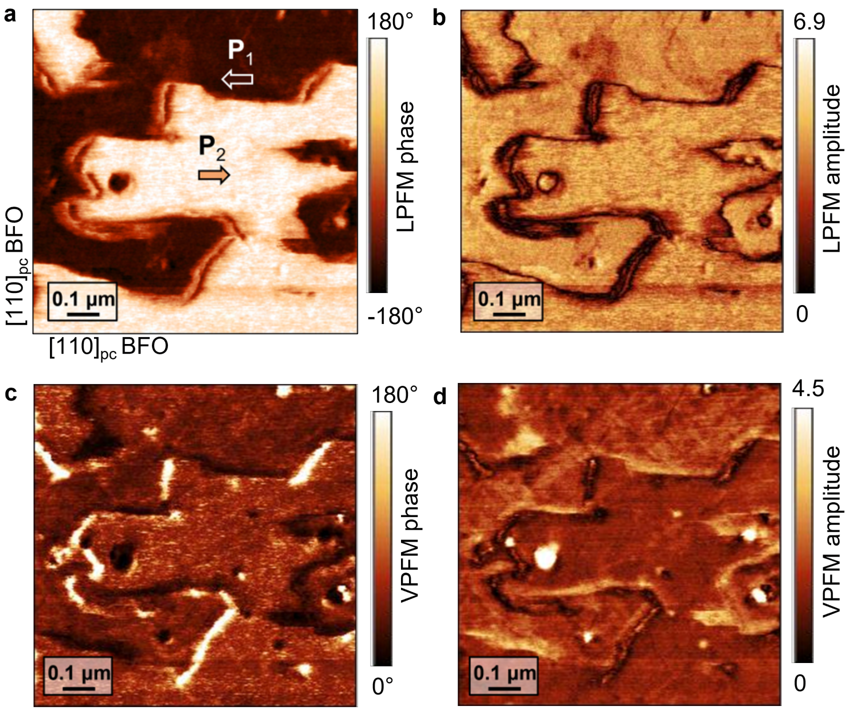

Supplementary Fig. 14. | LPFM and VPFM phase and amplitude contrast at the BFO domains and domain walls on BFTO|LSMO. LPFM (**a**) phase and (**b**) amplitude and VPFM (**c**) phase and (**d**) amplitude, corresponding to Fig. 4a,c in the main text.

Separated phase and amplitude channels of PFM images recorded for the HH and TT walls in the BFO film on BFTO|LSMO. Here we show separated phase and amplitude channels of LPFM and VPFM images recorded for the BFO|BFTO heterostructure (corresp. Fig. 4a,b of the main text). The uniaxial in-plane polarization in the BFO film on BFTO|LSMO results in the expected 180° domain contrast in the LPFM phase for the two different domain states, see Supplementary Fig. 14a. The minimum LPFM amplitude is observed at the walls (Supplementary Fig. 14b). The VPFM phase shows the deterministic out-of-plane contrast change at the tail-to-tail and head-to-head walls (Supplementary Fig. 14c). It corroborates the appearance of out-of-plane polarization components at the walls, that is, a gradual rotation of the electric-dipole moments across the wall rather than displaying Ising behavior. Specifically, each HH wall exhibits a downward polarized state, while each TT wall is associated with an upward polarization, suggesting homochirality of

the BFO domain walls. Lastly, the VPFM amplitude signal at the walls (Supplementary Fig. 14d) indicates a non-equivalent change of piezoresponse at the homochiral tail-to-tail and head-to-head walls. In particular, the amplitude of the out-of-plane polarization increases at the head-to-head walls and decreases at the tail-to-tail walls, in agreement with negative charges at the interface creating a preference for downward polarization, see Supplementary Note 1.

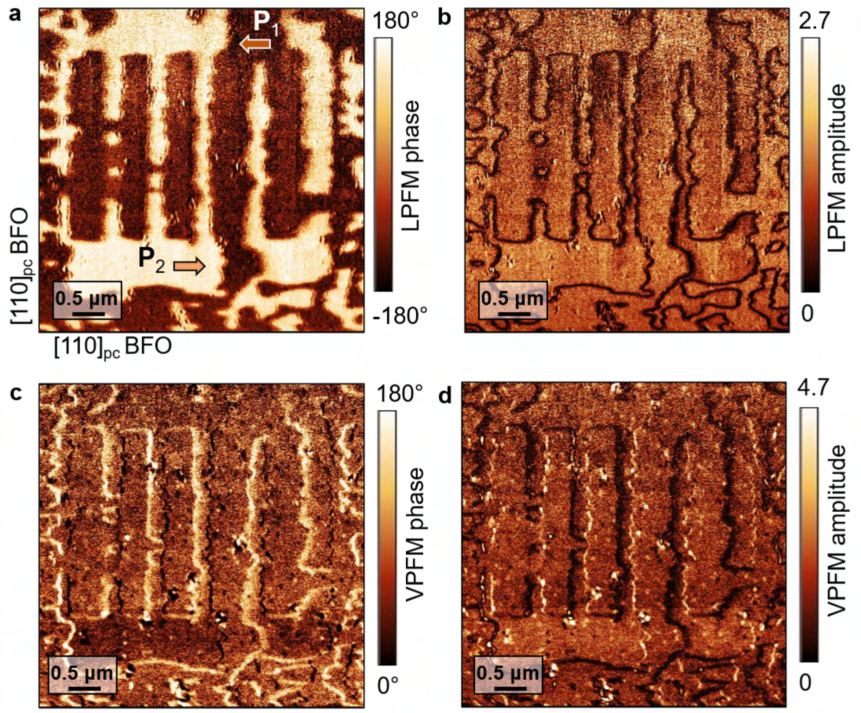

Supplementary Fig. 15. | LPFM and VPFM scans of the artificially created in-plane-polarized stripe domains and the corresponding chiral domain walls in BFO on BFTO|LSMO. LPFM (**a**) phase and (**b**) amplitude images show artificially created in-plane-polarized stripe domains in the uniformly downward-polarized BFO film. VPFM (**c**) phase and (**d**) amplitude images confirm that the written domain walls show the same vertical piezoresponse as the pristine domain walls (see Fig. 4 of the main text), and hence are also homochiral.

Electric-field-induced creation of chiral domain walls. In order to demonstrate the robustness of the chiral texture in BFO films grown on BFTO|LSMO|NGO, we performed local poling experiments and used the scanning-probe-tip-induced trailing field[19] to design artificial HH and TT domain walls. The polarization of the films was first set to an upward orientation and then switched back to the preferential downward orientation while trailing along the [010] axis of NGO. This enabled us to create in-plane-polarized stripe domains in the BFO film and conveniently visualize

alternating upward and downward-oriented polarization components at the TT and HH domain walls, respectively, thus corroborating the existence of 251° domain walls.

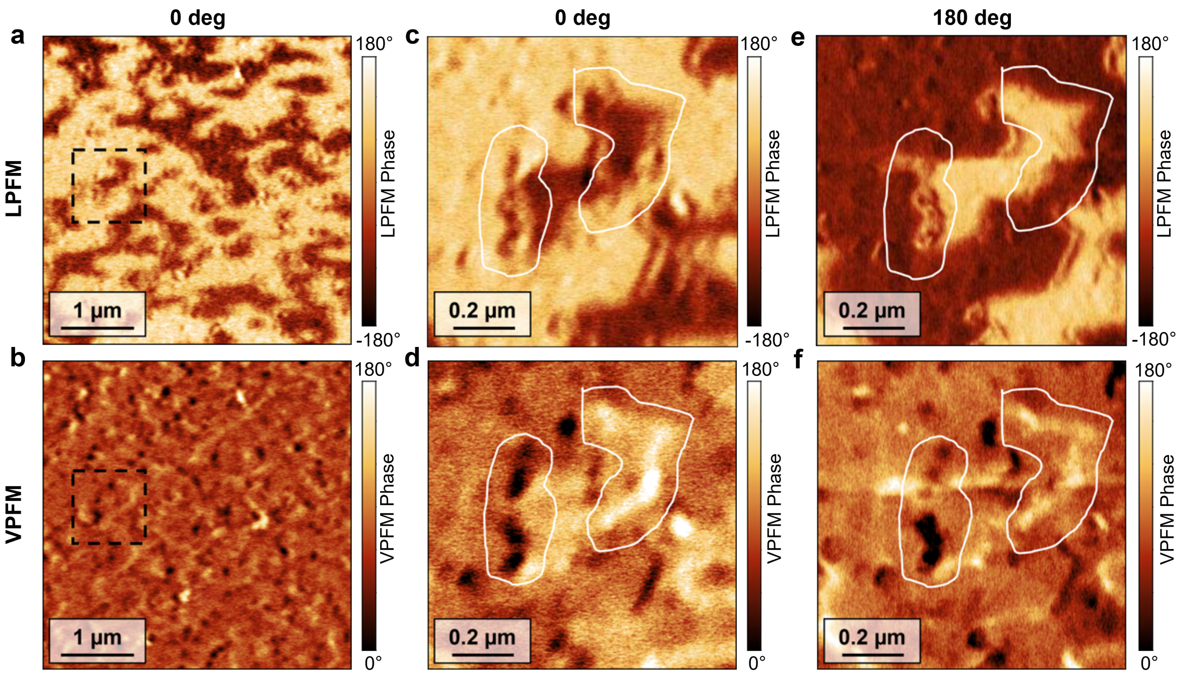

Supplementary Fig. 16. | Confirmation of out-of-plane polarization at the BFO domain walls on BFTO|LSMO using vector PFM. In order to distinguish the source of the observed discontinuities in the VPFM signal, we select a micron-sized area (marked in the large-area LPFM and VPFM phase scans (**a**, **b**, resp.) to resolve the domain walls. The high-resolution LPFM and VPFM scans are shown in **c** and **d**, respectively. Then the sample is rotated by 180° and the same area is rescanned. The LPFM signal (**e**) gets inverted, but the VPFM signal (**f**) at the walls does not (highlighted with a white line for convenience). This confirms that the signal originates from the cantilever deflection and not buckling, and hence implies a genuine out-of-plane polarization at the walls.

Out-of-plane polarization as the origin of the VPFM signal at the HH and TT walls. To confirm that the observed VPFM signals at the BFO domain walls are related to out-of-plane polarization, we perform PFM scans of the same area upon 180° sample rotation, as shown in Supplementary Fig. 16. We select a micron-sized area in the large-area LPFM and VPFM images (Supplementary Fig. 16a,b). Higher-resolution scans in this area reveal the piezoresponse signals consistent with the domain-wall homochirality (Supplementary Fig. 16c,d). We then rotate the sample by 180° and perform the same high-resolution scans. While the LPFM signal (Supplemen-

tary Fig. 16e) is inverted, the VPFM signal (Supplementary Fig. 16f) at the walls does not. This confirms that the signal originates from cantilever deflection and implies pure out-of-plane polarization localized at the walls. It additionally rules out the cantilever buckling due to additional in-plane polarization components at the walls (e.g. those perpendicular to the uniaxial in-plane polarization axis).

Supplementary Note 7. Excluding the possibility of artifacts in the PFM measurements of the homochiral BFO domain walls (incl. Supplementary Fig. 17-19)

The PFM investigation of domain walls in the BFO films deposited on BFTO|LSMO|NGO reveals unique signatures of homochirality, as shown in Fig. 4 of the main text. To confirm that the measured signals are intrinsic and not related to any spurious effects, in this section we exclude potential sources of artifacts:

1. Conduction at the charged 109° domain walls in the classical stripe BFO films is known to give rise to spurious piezoresponse signals. To rule out such local conduction as the origin of the observed VPFM signals, we performed conducting AFM (C-AFM) analysis on BFO|BFTO|LSMO, see Supplementary Fig. 17. The measured conduction at the non-Ising domain walls in our sample is weak and diffuse, therefore unlikely to affect our PFM measurements and cause the VPFM signals we observe at the domain walls.

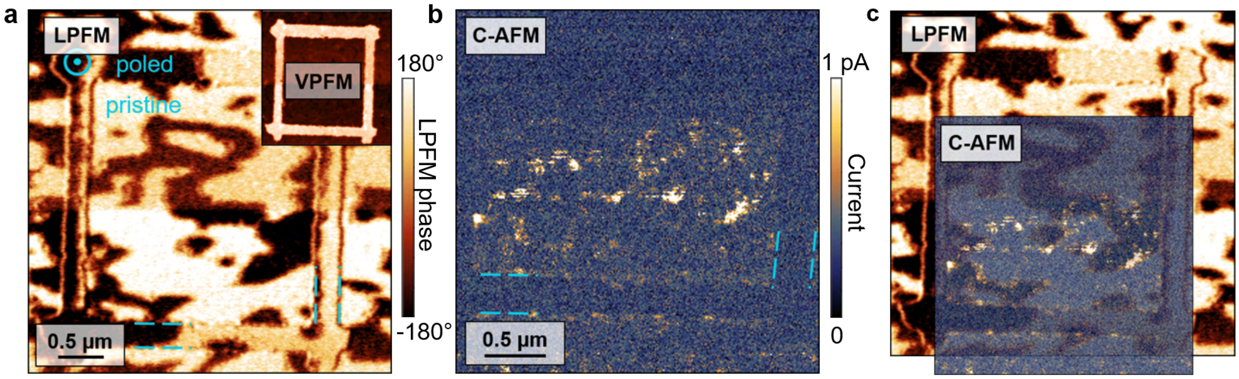

Supplementary Fig. 17. | Attenuated conduction at the homochiral BFO domain walls on BFTO|LSMO|NGO. a, The LPFM phase image of BFO domains in BFO|BFTO|LSMO. The artificially upward-poled frame (dashed blue contours, also visible in the VPFM phase, see inset) is used as a reference. **b**, C-AFM measurement performed in the corresponding area shows no significant conduction of the homochiral BFO walls compared to artificially poled 180° domain walls (dashed blue contours). **c**, C-AFM contrast overlaid with the LPFM image for easier identification of domain-wall-related conduction.

We additionally note that the deterministic alternation of the VPFM signal at our TT and HH walls is different from the reported observations of conduction-related vertical piezoresponse

that always has the same directionality, e.g. always appears as upward polarization in the downward polarized matrix[3].

2. We excluded the shear-strain-related artifacts that could appear when scanning perpendicular to domain walls[20] by exploring all possible scanning directions (i.e. from down to up, from left to right, etc.). The PFM contrast stays unchanged, see Supplementary Fig. 18, ruling out the possibility of such artifacts.

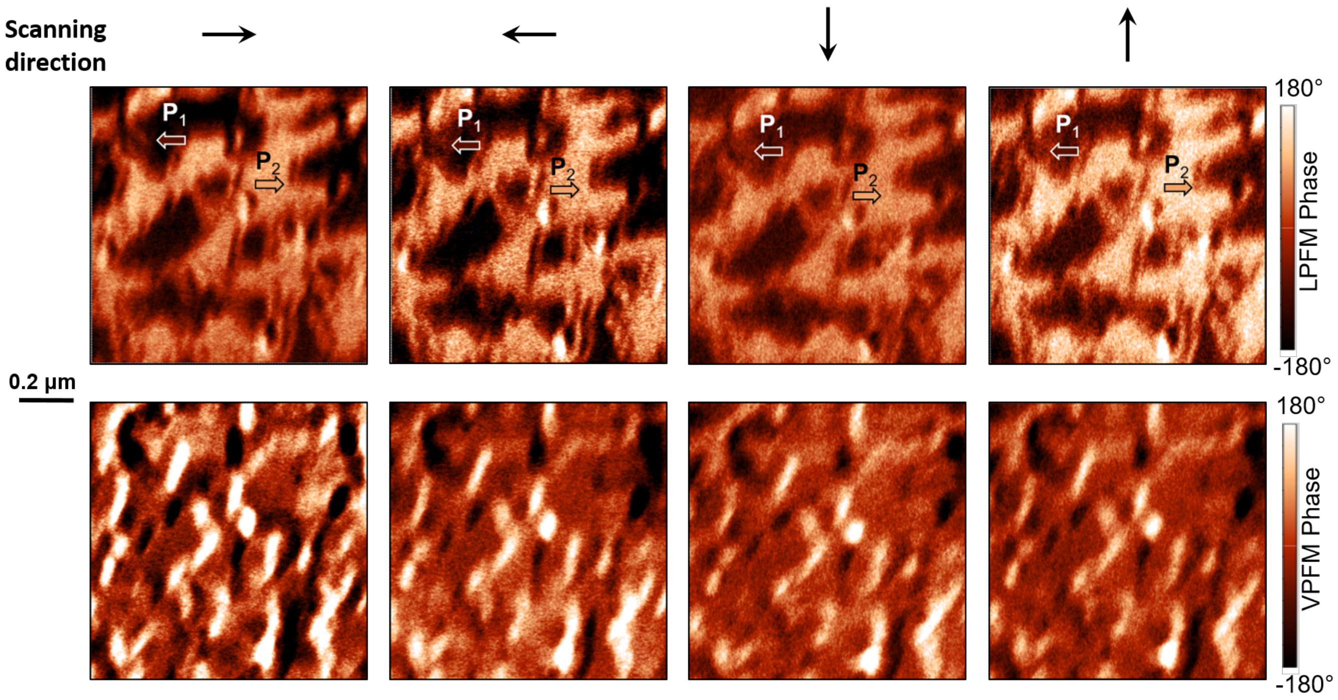

Supplementary Fig. 18. | Scanning direction independence of the VPFM signal at the BFO domain walls on BFTO|LSMO|NGO. The appearance of the bright VPFM signal for all the TT walls and the dark VPFM signal at all the HH walls regardless of the scanning direction rules out the possibility of shear-strain-related artifacts and confirms the intrinsic signal related to out-of-plane polarization directly at the walls.

3. The elastic response of the crystal to stress across charged domain walls has been previously reported to appear as the VPFM signal[21]. The vertical deflection we observe, however, is directed opposite to what could be expected from this consideration and, therefore, can be ruled out.

4. To further confirm the intrinsic nature of the measured PFM amplitude and phase signals, we show the contrast arising at the homochiral domain walls in the same scan with the expected contrast measured at the artificially poled 180° BFO domains, see Supplementary Fig. 19.

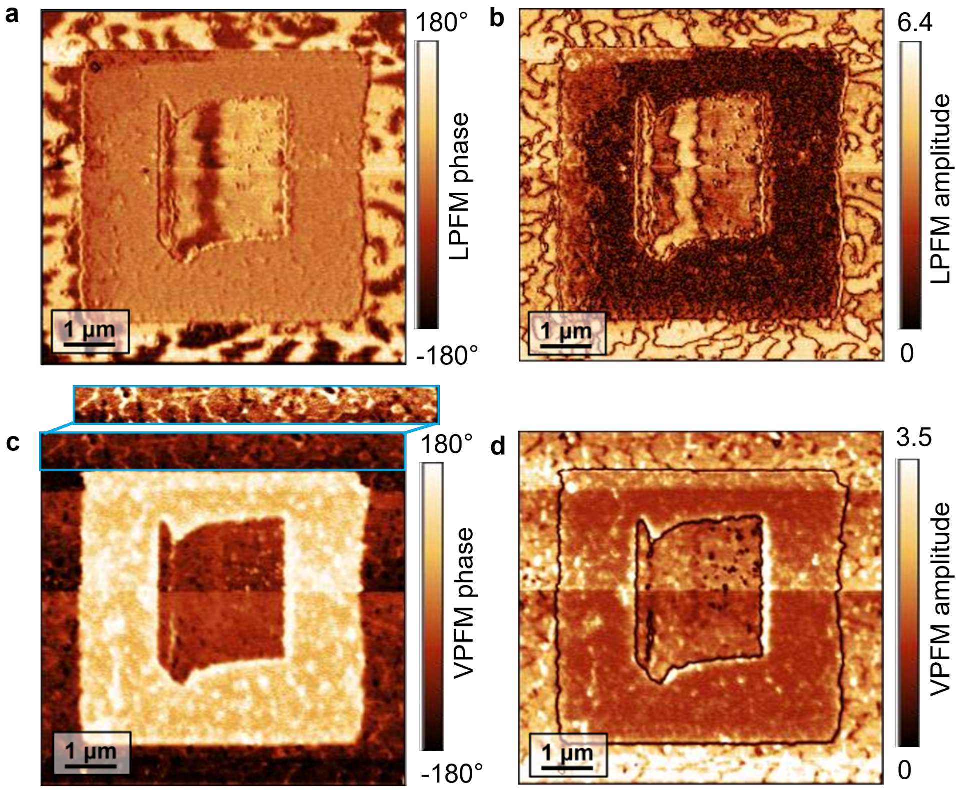

Supplementary Fig. 19. | Comparison of PFM contrast recorded for the homochiral (109° and 251°) BFO domain walls and artificially created 180° BFO domain walls in BFO|BFTO|LSMO. The LPFM (**a**) phase and (**b**) amplitude and the VPFM (**c**) phase and (**d**) amplitude images of BFO domain walls in BFO|BFTO|LSMO. Inset in **c** shows the indicated part of the pristine film with an adjusted scale bar to highlight the VPFM phase signal at the homochiral BFO walls.

5. We performed STEM investigations to confirm the unconventional 251° rotation of the electric dipoles at the BFO domain walls on BFTO|LSMO. Fig. 4i in the main text shows a segment of one of the 251° tail-to-tail (TT) domain walls, which extends over tens of nanometers. The regions marked by the block arrows clearly support the 251° nature of the domain wall, which is in line with the polarization rotation expected for the TT wall, as sketched in Fig. 4h of the main text. Hence, the agreement between the STEM and PFM

measurements further supports the statement of the domain-wall homochirality.

Supplementary Note 8. Excluding epitaxial strain as the sole origin for the homochiral BFO domain walls (incl. Supplementary Fig. 20)

We verified that the formation of homochiral BFO domain walls cannot be explained by the epitaxial strain and related flexoelectric effects by investigating BFO films grown directly on LSMO (20 u.c.)|NGO, without inserting the BFTO buffer. Supplementary Fig. 20 reveals that no signatures of the non-Ising polarization textures can be detected in these heterostructures. This helps to associate the observed homochirality of the BFO domain walls uniquely to the Aurivillius buffer layer.

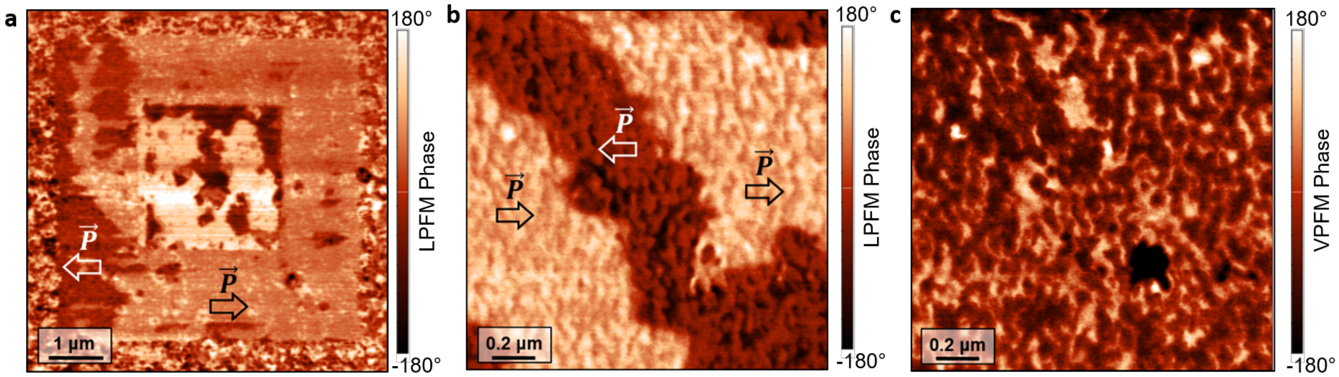

Supplementary Fig. 20. | PFM study of switched ferroelectric domains in BFO|LSMO|NGO confirming the absence of non-Ising domain walls. **a**, LPFM scan of the BFO film on LSMO|NGO. The pristine BFO film exhibits a formation of 180° out-of-plane-polarized nanodomains (visible outside of the reversibly poled area). No stabilization of charged domain walls is detected. Larger in-plane domains can be temporarily stabilized upon the local electrical poling. We confirm that these domains exhibit uniaxial in-plane polarization, similar to Supplementary Fig. 13. Higher-magnification (**b**) LPFM and (**c**) VPFM scans of artificially formed HH and TT walls show no signatures of Néel-type chiral domain walls in this film, confirming that the BFTO buffer layer creates the symmetry-breaking required for the net chirality in the BFO domain walls to emerge.

Supplementary Note 9. Phase field simulation of the homochiral BFO domain walls (incl. Supplementary Table 2 and Supplementary Fig. 21)

The chirality of a domain wall can be judged by evaluating a Lifschitz invariant term $P_x \nabla P_z - \nabla P_x P_z$ [22]. In most ferroelectric materials that host Néel-type walls, left-handed and right-handed walls are equally common. However, due to structural Dzyaloshinskii-Moriya interactions[23] one chirality can become favored over the other, for which the above-mentioned term enters the free energy expansion. We start with the Landau Free-energy expansion of a cubic ferroelectric material with three possible polarization directions, applicable to $BiFeO_3$:

$$F = \frac{a}{2}(P_x^2 + P_y^2 + P_z)^2 + \frac{b}{4}(P_x^2 + P_y^2 + P_z^2) + \frac{c}{4}(P_x^4 + P_y^4 + P_z^4) \quad , \tag{4}$$

where x, y, z correspond to the pseudocubic axes. From our experiments we know, that the only polarization axes that are observed are $[110]_{\text{pc}}$ and $[001]_{\text{pc}}$. We thus perform a 45° rotation in the x-y-plane of our coordinate system, in order to align P_x with $[110]_{\text{pc}}$ and P_y with $[1\bar{1}0]_{\text{pc}}$. Since the latter polarization direction is not observed, we then set it to zero, allowing us to reduce the phase space to the polarization directions P_x ($[110]_{\text{pc}}$) and P_z ($[001]_{\text{pc}}$). The new free energy expression reads:

$$F = \frac{a}{2}(P_x^2 + P_z^2) + \frac{b}{4}(P_x^2 + P_z^2)^2 + \frac{c}{8}(P_x^4) + \frac{c}{4}P_z^4 \tag{5}$$

$$+ \sigma(P_x \nabla P_z - \nabla P_x P_z) \tag{6}$$

$$- E_x P_x - E_z P_z \tag{7}$$

where a, b, c are the Landau expansion parameters of the polar mode. We included the Lifshitz invariant with amplitude σ and the last two terms describe the coupling to an electric field. We use the Landau parameters shown in Supplementary Table 2, to qualitatively reproduce the behavior of the BFO in the BFO|BFTO heterostructure. These parameters reproduce an absolute polarization

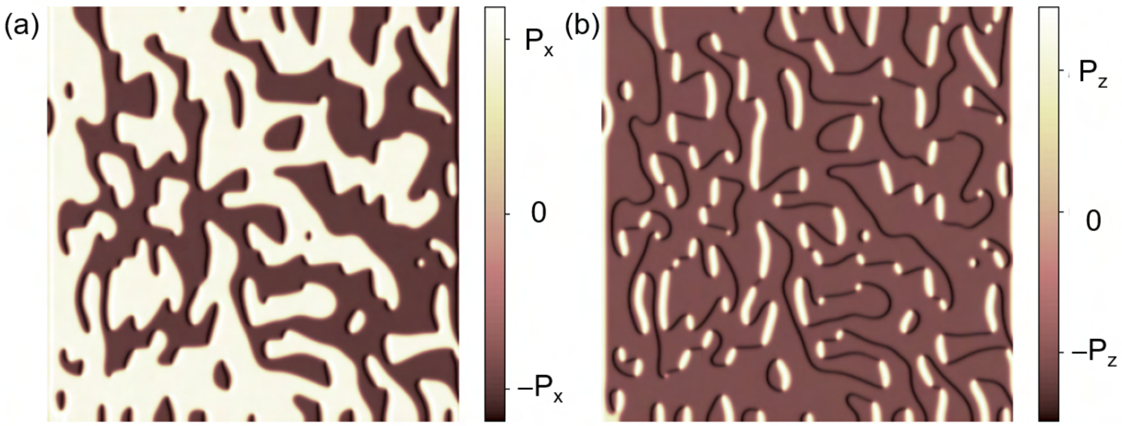

Supplementary Fig. 21. | Phase field simulation of chiral domain walls in systems with non-zero Lifshitz invariant. (**a**) The simulated in-plane-polarized domains and (**b**) the out-of-plane polarization emergence at the domain walls are comparable to the measured LPFM and VPFM phase scans of BFO|BFTO system, respectively (see Figure 4a,b of the main text).

of $P_x = \sqrt{2}P_z$ at the 4 global minima at $[111]_{\mathrm{pc}}$, $[\bar{1}\bar{1}1]_{\mathrm{pc}}$, $[11\bar{1}]_{\mathrm{pc}}$ or $[\bar{1}\bar{1}\bar{1}]_{\mathrm{pc}}$, i.e. the uniaxial in-plane polarization in BFO we observe experimentally. In addition, we apply an electric field E_z along $-z$ to simulate the field arising due to the interfacial bound charge. This reduces the number of stable polarization states from four to two, $[11\bar{1}]_{\mathrm{pc}}$ and $[\bar{1}\bar{1}\bar{1}]_{\mathrm{pc}}$ that are downward polarized, in accordance with our experimental observations. We solve the Euler equations resulting from (7) using the PDE solvers implemented in the FiPy package[24]. The resulting phase field simulation shown in Supplementary Fig. 21 reproduces our experimental PFM data (see Fig. 4a,b of the main text) and confirms that the homochirality of domain walls in the BFO films grown on BFTO can be emulated by considering a ferroelectric DMI-like interaction[23,25].

a	b	c	σ
-1.	2.	2.	0.35

Supplementary Table 2. Landau parameters used for the phase field simulation.

References

1. Spaldin, N. A., Efe, I., Rossell, M. D. & Gattinoni, C. Layer and spontaneous polarizations in perovskite oxides and their interplay in multiferroic bismuth ferrite. *The Journal of Chemical Physics* **154**, 154702 (2021).

2. Gradauskaite, E., Gray, N., Campanini, M., Rossell, M. D. & Trassin, M. Nanoscale Design of High-Quality Epitaxial Aurivillius Thin Films. *Chemistry of Materials* **33**, 9439–9446 (2021).

3. Campanini, M., Trassin, M., Ederer, C., Erni, R. & Rossell, M. D. Buried In-Plane Ferroelectric Domains in Fe-Doped Single-Crystalline Aurivillius Thin Films. *ACS Applied Electronic Materials* **1**, 1019–1028 (2019).

4. Yu, P., Luo, W., Yi, D., Zhang, J. X., Rossell, M. D., Yang, C.-H., You, L., Singh-Bhalla, G., Yang, S. Y., He, Q., Ramasse, Q. M., Erni, R., Martin, L. W., Chu, Y. H., Pantelides, S. T., Pennycook, S. J. & Ramesh, R. Interface control of bulk ferroelectric polarization. *Proceedings of the National Academy of Sciences* **109**, 9710–9715 (2012).

5. Resta, R. & Vanderbilt, D. *Theory of Polarization: A Modern Approach*, 31–68 (Springer Berlin Heidelberg, 2007).

6. King-Smith, R. D. & Vanderbilt, D. Theory of polarization of crystalline solids. *Phys. Rev. B* **47**, 1651–1654 (1993).

7. Stengel, M. & Vanderbilt, D. Berry-phase theory of polar discontinuities at oxide-oxide interfaces. *Phys. Rev. B* **80**, 241103 (2009).

8. Vasylechko, L., Akselrud, L., Morgenroth, W., Bismayer, U., Matkovskii, A. & Savytskii, D. Crystal structure of $NdGaO_3$ at 100 K and 293 K based on synchrotron data. *Journal of Alloys and Compounds* **297**, 46–52 (2000).

9. Buttner, R. H. & Maslen, E. N. Structural parameters and electron difference density in $BaTiO_3$. *Acta Crystallographica Section B* **48**, 764–769 (1992).

10. Kubel, F. & Schmid, H. Structure of a ferroelectric and ferroelastic monodomain crystal of the perovskite $BiFeO_3$. *Acta Crystallographica Section B* **46**, 698–702 (1990).

11. Hervoches, C. H., Snedden, A., Riggs, R., Kilcoyne, S. H., Manuel, P. & Lightfoot, P. Structural behavior of the four-layer aurivillius-phase ferroelectrics $SrBi_4Ti_4O_{15}$ and $Bi_5Ti_3FeO_{15}$. *Journal of Solid State Chemistry* **164**, 280–291 (2002).

12. Boschker, H., Mathews, M., Houwman, E. P., Nishikawa, H., Vailionis, A., Koster, G., Rijnders, G. & Blank, D. H. Strong uniaxial in-plane magnetic anisotropy of (001)- and (011)-oriented $La_{0.67}Sr_{0.33}MnO_3$ thin films on $NdGaO_3$ substrates. *Physical Review B - Condensed Matter and Materials Physics* **79**, 214425 (2009).

13. Damodaran, A. R., Breckenfeld, E., Chen, Z., Lee, S. & Martin, L. W. Enhancement of Ferroelectric Curie Temperature in $BaTiO_3$ Films via Strain-Induced Defect Dipole Alignment. *Advanced Materials* **26**, 6341–6347 (2014).

14. Strkalj, N., De Luca, G., Campanini, M., Pal, S., Schaab, J., Gattinoni, C., Spaldin, N. A., Rossell, M. D., Fiebig, M. & Trassin, M. Depolarizing-Field Effects in Epitaxial Capacitor Heterostructures. *Physical Review Letters* **123**, 147601 (2019).

15. De Luca, G., Strkalj, N., Manz, S., Bouillet, C., Fiebig, M. & Trassin, M. Nanoscale design of polarization in ultrathin ferroelectric heterostructures. *Nature Communications* **8**, 1419 (2017).

16. Gradauskaite, E., Hunnestad, K. A., Meier, Q. N., Meier, D. & Trassin, M. Ferroelectric Domain Engineering Using Structural Defect Ordering. *Chemistry of Materials* **34**, 6468–6475 (2022).

17. Keeney, L., Saghi, Z., O'Sullivan, M., Alaria, J., Schmidt, M. & Colfer, L. Persistence of Ferroelectricity Close to Unit-Cell Thickness in Structurally Disordered Aurivillius Phases. *Chemistry of Materials* **32**, 10511–10523 (2020).

18. Zu, R., Wang, B., He, J., Wang, J. J., Weber, L., Chen, L. Q. & Gopalan, V. Analytical and numerical modeling of optical second harmonic generation in anisotropic crystals using SHAARP package. *npj Computational Materials* **8**, 1–12 (2022).

19. Sluka, T., Tagantsev, A. K., Damjanovic, D., Gureev, M. & Setter, N. Enhanced electromechanical response of ferroelectrics due to charged domain walls. *Nature Communications* **3**, 748 (2012).

20. Guyonnet, J., Béa, H., Guy, F., Gariglio, S., Fusil, S., Bouzehouane, K., Triscone, J. M. & Paruch, P. Shear effects in lateral piezoresponse force microscopy at 180° ferroelectric domain walls. *Applied Physics Letters* **95**, 132902 (2009).

21. Lochocki, E. B., Park, S., Lee, N., Cheong, S. W. & Wu, W. Piezoresponse force microscopy of domains and walls in multiferroic $HoMnO_3$. *Applied Physics Letters* **99**, 232901 (2011).

22. Houchmandzadeh, B., Lajzerowicz, J. & Salje, E. Order parameter coupling and chirality of domain walls. *Journal of Physics: Condensed Matter* **3**, 5163–5169 (1991).

23. Zhao, H. J., Chen, P., Prosandeev, S., Artyukhin, S. & Bellaiche, L. Dzyaloshinskii–Moriya-like interaction in ferroelectrics and antiferroelectrics. *Nature Materials* **20**, 341–345 (2021).

24. Guyer, J. E., Wheeler, D. & Warren, J. A. Fipy: Partial differential equations with python. *Computing in Science Engineering* **11**, 6–15 (2009).

25. Erb, K. C. & Hlinka, J. Vector, bidirector, and Bloch skyrmion phases induced by structural crystallographic symmetry breaking. *Physical Review B* **102**, 024110 (2020).
